# Enhancing Electrochemical Water-Splitting Kinetics by Polarization-Driven Formation of Near-Surface Iron(0): An In Situ XPS Study on Perovskite-Type Electrodes**

**DOI:** 10.1002/anie.201409527

**Published:** 2014-12-30

**Authors:** Alexander K Opitz, Andreas Nenning, Christoph Rameshan, Raffael Rameshan, Raoul Blume, Michael Hävecker, Axel Knop-Gericke, Günther Rupprechter, Jürgen Fleig, Bernhard Klötzer

**Affiliations:** Vienna University of Technology, Institute of Chemical Technologies and AnalyticsGetreidemarkt 9/164-EC, 1060 Vienna (Austria); University of Innsbruck, Institute of Physical ChemistryInnrain 80-82, 6020 Innsbruck (Austria); Vienna University of Technology, Institute of Materials ChemistryGetreidemarkt 9/165-PC, 1060 Vienna (Austria); Fritz Haber Institute of the Max Planck Society, Department of Inorganic ChemistryFaradayweg 4–6, 14195 Berlin (Germany); Catalysis for Energy, Group E-GKAT, Helmholtz-Zentrum Berlin fuer Materialien und Energie GmbH, Division Solar Energy ResearchElektronenspeicherring BESSY II, Albert-Einstein-Strasse 15, 12489 Berlin (Germany)

**Keywords:** electrocatalysis, heterogeneous catalysis, perovskites, solid oxide electrolysis cells, thin-film electrodes

## Abstract

In the search for optimized cathode materials for high-temperature electrolysis, mixed conducting oxides are highly promising candidates. This study deals with fundamentally novel insights into the relation between surface chemistry and electrocatalytic activity of lanthanum ferrite based electrolysis cathodes. For this means, near-ambient-pressure X-ray photoelectron spectroscopy (NAP-XPS) and impedance spectroscopy experiments were performed simultaneously on electrochemically polarized La_0.6_Sr_0.4_FeO_3−*δ*_ (LSF) thin film electrodes. Under cathodic polarization the formation of Fe^0^ on the LSF surface could be observed, which was accompanied by a strong improvement of the electrochemical water splitting activity of the electrodes. This correlation suggests a fundamentally different water splitting mechanism in presence of the metallic iron species and may open novel paths in the search for electrodes with increased water splitting activity.

Solid oxide electrochemical cells are highly promising devices for efficient conversion of chemical into electrical energy (solid oxide fuel cells, SOFCs) as well as vice versa (solid oxide electrolysis cells, SOECs). SOFCs are already commercialized, and a Ni/yttria stabilized zirconia (YSZ) ceramic–metal composite (cermet) is successfully employed as anode material.[1] In contrast, SOECs are still lagging behind, and often SOFCs are simply used in “reverse mode” for running electrolysis, which may cause severe degradation of the Ni/YSZ electrode.[2,3] A possible solution is to replace Ni/YSZ by mixed ionic and electronic conductors (MIEC). MIECs are already used as electrodes in an oxidizing atmosphere, and mixed conducting oxides such as La_0.6_Sr_0.4_FeO_3−*δ*_ (LSF) are also interesting candidates for future SOEC cathodes, as large parts of the electrode surface may become electrochemically active[4–8] rather than just the triple phase-boundary region as in Ni/YSZ.[9,10] However, little is known about the surface chemistry of mixed conducting perovskite-type electrodes in a reducing atmosphere and its relation to the electrochemical properties. Especially knowledge on the changes in near-surface composition and cation valence states upon electrochemical polarization is rare.

Near-ambient pressure X-ray photoelectron spectroscopy (NAP-XPS) is a powerful method for the investigation of MIECs under electrochemical polarization, providing information on adsorbed species as well as on near-surface cations of a polarized electrode.[11–13] In situ NAP-XPS studies were performed on perovskite-type electrodes in an oxidizing atmosphere[14–16] and on ceria-based electrodes under reducing conditions.[17–19] However, establishing a well-defined polarization state of a perovskite-type mixed conducting electrode in H_2_/H_2_O atmosphere and simultaneously probing the electrochemical surface activity as well as performing NAP-XPS measurements has to the best of our knowledge not been achieved yet. Especially the attainment of a well-defined and rather homogeneous polarization of the LSF electrode (despite being a poor electronic conductor under reducing conditions) allows the relation of changes in the electrochemical surface activity to the observed changes in surface chemistry.

Herein, we describe how synchrotron-based NAP-XPS and impedance spectroscopy were performed simultaneously, enabling the investigation of changes in surface chemistry and in water splitting kinetics of the perovskite-type mixed conductor La_0.6_Sr_0.4_FeO_3−*δ*_ (LSF) under electrochemical polarization in humid H_2_ atmospheres. An issue complicating such measurements on LSF is its low electronic conductivity under reducing conditions.[20,21] This can be overcome by a micro-structured metallic thin film, which acts as a current collector and may provide virtually homogeneous polarization of the working electrode[8,22] and thus a homogeneous driving force of the electrochemical surface reaction (see also the Supporting Information). Here, homogeneous polarization was realized by interdigitated Pt fingers buried beneath the 200 nm LSF layer. This model-composite working electrode was deposited on single-crystalline yttria stabilized zirconia (YSZ) with a porous counter electrode (Figure 1). Details on the preparation of LSF working electrodes with embedded current collectors and of porous counter electrodes are given in the Supporting Information.

**Figure 1:**
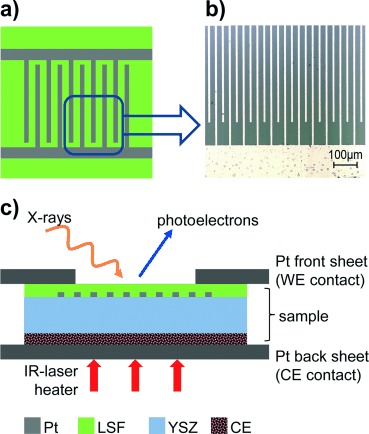
a) Sketch and b) optical micrograph of a sample with thin film LSF electrode and buried current collector. c) Sketch (cross section) of a sample mounted for NAP-XPS measurements (WE—working electrode, CE—counter electrode).

NAP-XPS measurements were conducted at the ISISS beam line of synchrotron HZB/BESSY II in Berlin.[23] NAP-XPS experiments under electrochemical polarization were realized by mounting the samples between two platinum sheets (top one with a hole), which served as mechanical fixation and as electrical contacts of the two electrodes (Figure 1 c). This novel setup allows a simultaneous measurement of the electrochemical reaction rate and surface activity (by dc current and impedance spectroscopy, respectively) as well as of the chemical surface composition and the near-surface cation valence states (by means of XPS; for experimental details, see the Supporting Information).

The measured current–overpotential characteristics of an LSF working electrode is shown in Figure 2. Three selected Fe 2p spectra, corresponding to three different polarization states, are depicted as insets and reveal that a metallic iron species already evolves at relatively low cathodic overpotentials. Upon formation of this Fe^0^ species, the electrochemical water splitting activity of the LSF surface strongly increased, leading to a highly asymmetric current–voltage curve, which does not follow an exponential function (for example, the Butler–Volmer equation; Supporting Information, Figure S1) with electrochemically meaningful parameters. This indicates a mechanistic change of the reaction kinetics of H_2_O+2 e^−^⇌H_2_+O^2−^ at the LSF surface. The cathodically formed Fe^0^ was quickly re-oxidized after removing the polarization (within the time between two simultaneous XPS/impedance measurements of about 200 to 600 s). Accordingly, the current–voltage curve in Figure 2 is highly reversible. Owing to this reversibility, an observation of the cathodically formed metallic Fe species is clearly only possible by means of in situ experiments as employed in the present study.

**Figure 2:**
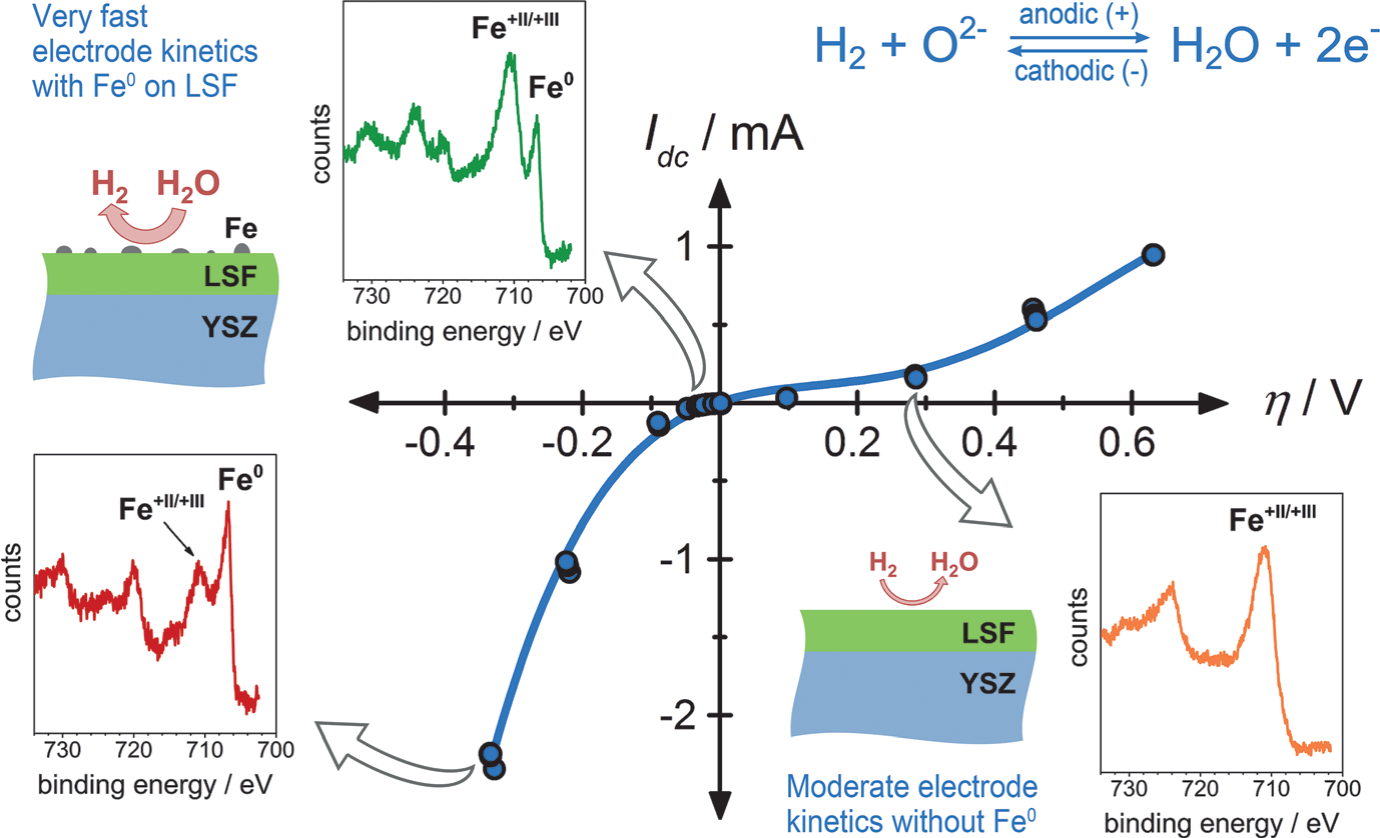
Current–overpotential curve (*I*_dc_ vs. *η*) of LSF in a humid reducing atmosphere (0.25 mbar H_2_+0.25 mbar H_2_O). The symbols represent measured values; the line is not a fit but a guide for the eye. The reaction proceeding on the surface of the LSF working electrode is given top right. For selected points of the curve (indicated by arrows), Fe 2p XPS spectra are shown as insets. The sketches indicate the situation for the LSF surface and the resulting reactivity, respectively.

At each point of the current–voltage curve (that is, for distinct reaction rates), NAP-XPS and impedance measurements were performed simultaneously. The main results of the impedance measurements are as follows (details regarding data analysis are given in the Supporting Information): From the recorded impedance spectra (compare with the Supporting Information, Figure S2), a surface resistance (*R*_surface_) was extracted. The area (*A*)-related inverse surface resistance 1/(*R*_surface_ *A*), which is a measure of the electrochemical activity of the LSF surface for the water splitting/hydrogen oxidation reaction, is plotted versus the overpotential *η* in Figure 3 a. In this plot, the strong asymmetry (especially at small overpotentials) is even more clearly visible: A strong anodic overpotential (*η*=+285 mV) causes an increase of 1/(*R*_surface_ *A*) by a factor of about three to four, whereas already a small cathodic polarization (*η*=−47 mV) leads to approximately the same change. A 70 mV increase of the cathodic overpotential (from −20 to −90 mV) improves the surface activity, quantified by 1/(*R*_surface_ *A*), by about one order of magnitude. In Figure 3 a, this strong asymmetry is reflected by an almost step-like non-linearity of the water-splitting activity in the cathodic regime and finds its counterpart in the XPS results, see below.

**Figure 3:**
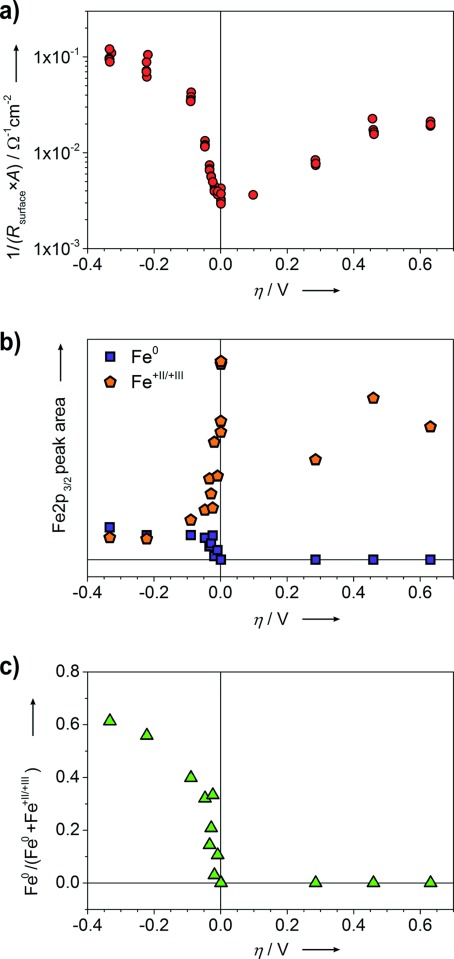
a) Area-related inverse surface resistance from impedance spectra vs. overpotential *η*. b) Fe 2p_3/2_ peak areas of the different Fe species (obtained by fits of XPS spectra) as a function of *η*. c) Fraction of Fe^0^ calculated from the peak area values in (b) plotted vs. overpotential.

The measured Fe 2p XPS spectra were analyzed using a simplified peak model with the main objective to quantify the relative proportions of metallic and oxidic iron and thus only the Fe 2p_3/2_ peak was considered in the fit (for details, see the Supporting Information, Section 3.3). The resulting peak areas of both Fe species (metallic and oxidic) and the fraction of Fe^0^ (with respect to the total Fe amount) are plotted versus the overpotential *η* in Figure 3 b and c, respectively. The amount and fraction of metallic iron abruptly increases for cathodic polarizations exceeding ca. −20 mV. In the same voltage range, the water splitting activity of the LSF electrode (that is, 1/(*R*_surface_ *A*)) also increases strongly. This correlation suggests a fundamental difference in the water-splitting mechanism on LSF with and without Fe^0^.

From the available data the exact structural morphology of the Fe^0^ species cannot be identified yet. Surface iron might be reduced to an atomic species while still being part of the perovskite lattice, but formation of metallic iron particles on the LSF surface appears more likely. The formation of a Fermi-edge in the valence band spectra (Supporting Information, Figure S3b) strongly indicates formation of a metallic phase and thus strongly suggests the latter mechanism; that is, the electrochemically driven evolution of metallic Fe particles on the LSF surface sketched in Figure 2. This is also supported by the observation that the total iron peak area (oxidic plus metallic) strongly decreases upon formation of Fe^0^ (Figure 3 b; Supporting Information, Figure S3a). Assuming particles being significantly larger than the mean free path of photoelectrons (which is ca. 0.5 nm), a significant amount of near-surface iron would become inaccessible for XPS measurements after Fe^0^ particle formation. Evolution of metallic iron particles already for small cathodic polarization is also in agreement with the fact that under these experimental conditions LSF is rather close to its thermodynamic stability limit.[21] Moreover, chemically driven formation of metal particles from perovskite-type SOFC anode materials under reducing conditions as well as the fully reversible reintegration of these particles under oxidizing conditions was reported recently.[24,25] Similar chemically driven formation of metallic Pd particles on Pd-doped LaFeO_3_ catalysts under reducing conditions was also found.[26] The observed stability of the LSF electrodes under reducing conditions may be due to the near-surface formation of Ruddlesden–Popper like phases upon Fe^0^ particle evolution.[24] High chemical stability of such phases in hydrogen atmosphere was reported for Cr/Ni containing Ruddlesden–Popper oxides.[27]

The strongly enhanced electrochemical kinetics even for small cathodic overpotentials may be caused by the presence of Fe^0^ particles at the surface promoting the kinetics of the water splitting reaction. However, also the remaining Fe-depleted oxide surface (of unknown crystal structure) may offer significantly faster electrode kinetics; for example, by a drastically changing Fe^+II^/Fe^+III^ ratio at the oxide surface upon Fe^0^ formation. Furthermore, a complex mechanistic interplay of both a metal and an oxide phase is conceivable; two-phase promoted electrochemical kinetics has been reported for perovskite-type electrodes in oxygen.[14,28,29]

Only further measurements can reveal details regarding the Fe^0^ containing phase, the rather complicated mechanistic reasons for the uncommon *I*–*V* curve, and the possibilities to realize such highly active SOEC cathodes also by chemical preparation rather than by polarization. In any case, the measured almost steplike improvement of the electrode performance by Fe^0^ exsolution emphasizes the high complexity of such a system but provides new directions in the search of novel oxide based electrodes for high-temperature electrochemical water splitting.
