# Supplementary material for: Enhancing Electrochemical Water-Splitting Kinetics by Polarization-Driven Formation of Near-Surface Iron(0): An In Situ XPS Study on Perovskite-Type Electrodes
Source: Angew Chem Int Ed Engl. 2014 Dec 30;54(9):2628–32. doi: 10.1002/anie.201409527 (PMC4506551; doi:10.1002/anie.201409527)
Supplement: Supplementary file 1 [file anie0054-2628-sd1.pdf]

Angewandte Chemie
Eine Zeitschrift der Gesellschaft Deutscher Chemiker

Supporting Information

© Wiley-VCH 2015

69451 Weinheim, Germany

Enhancing Electrochemical Water-Splitting Kinetics by Polarization-Driven Formation of Near-Surface Iron(0): An In Situ XPS Study on Perovskite-Type Electrodes**

Alexander K. Opitz, Andreas Nenning, Christoph Rameshan, Raffael Rameshan, Raoul Blume, Michael Hävecker, Axel Knop-Gericke, Günther Rupprechter, Jürgen Fleig, and Bernhard Klötzer*

anie_201409527_sm_miscellaneous_information.pdf

1. Experimental

1.1 Sample preparation

The LSF working electrodes with buried platinum current collectors were prepared as follows: 5 nm Ti (BAL-TEC, Germany) and 100 nm Pt (99.95 % pure, OEGUSSA, Austria) were sputter deposited onto yttria-stabilized zirconia (single crystals, (100)-oriented, 9.5 mol% Y_2O_3; supplier: CrysTec, Germany) and micropatterned by subsequent photolithography and argon ion-beam etching. Thin films of LSF were applied on top of the current collector by pulsed laser deposition (PLD) with a 248 nm Kr-F-excimer laser (Compex Pro 201F, Coherent Lambda, Germany); primary laser energy and repetition rate were 400 mJ and 5 Hz, respectively. The oxygen background pressure in the PLD chamber was 4×10^{-2} mbar, substrate temperature was 650 °C (measured by pyrometer, Heitronics, Germany), and the deposition time of 30 min lead to a film thickness of about 300 nm. The resulting active areas of the LSF thin film electrodes were 0.111 cm^2. The LSF target was prepared from $La_{0.6}Sr_{0.}4FeO_{3-\delta}$ powder (Sigma-Aldrich) by isostatic pressing and sintering at 1200 °C. A sketch and a micrograph of an LSF working electrode with buried current collector are depicted in the main text in Fig. 1a and b, respectively.

The porous counter electrodes were fabricated prior to the working electrode preparation by subsequent application of LSF paste ($La_{0.6}Sr_{0.4}FeO_{3-\delta}$ powder, ethyl cellulose, and α-Terpineol; all Sigma-Aldrich) and Pt paste (Gwent Electronics, UK), followed by drying and five hour annealing at 850 °C. It should be emphasized that the polarization resistance of the counter electrode was measured to be approx. 8 Ω at 620°C, which is significantly smaller than the resistance of the thin film working electrode (see below). Consequently, a voltage drop at the counter electrode can safely be neglected (i.e. the porous electrode acts as both counter electrode and reversible reference electrode). The polarization of the working electrode can therefore be deduced from two-wire measurements by subtracting the ohmic voltage drop in the YSZ electrolyte (and the contacting wires) from the set voltage; details regarding the calculation of the overpotential are given in Sec. 2.1.

1.2 Near-ambient pressure XPS experiments and simultaneous impedance spectroscopy measurements under electrochemical polarization

The experiments were performed at the ISISS beam line of the HZB/BESSY II synchrotron in Berlin[1] with the new near-ambient pressure high energy XPS setup (NAP-HE-XPS). This setup enables XPS and XAS measurements at elevated pressures (up to 20 mbar; typically

1 mbar) in a continuous range from soft to hard X-rays. The main parts are a high pressure cell with an attached differentially pumped hemispherical analyzer (Phoibos 150 Plus, SPECS) including a 2D delay line detector. General as well as more detailed description of the near-ambient pressure XPS-setup is given in Refs. [2-4].

A sketch of a sample, which was used for in-situ XPS experiments under electrochemical polarization and simultaneous impedance measurements, is given in Fig. 1c of the main text. The LSF working electrode was connected with the positive pin of the impedance analyzer/potentiostat, the porous counter electrode with the grounded contact. The samples were heated via the Pt back sheet using an infrared laser. The temperature was controlled by means of a pyrometer (IMPAC IGAR12-LO) measuring the MIEC surface temperature, as well as by the conductivity of the YSZ electrolyte obtained from electrochemical impedance measurements[5, 6] Both methods consistently yielded temperatures of 620 ± 15 °C. Electrochemical impedance measurements with and without dc bias were carried out by an Alpha-A high performance frequency analyzer equipped with a POT/GAL 30V 2A interface (both: Novocontrol, Germany). Impedance spectroscopy was performed in a frequency range between 10 mHz and 1 MHz and the ac root mean square voltage was limited to 5 mV to avoid XPS peak broadening.

For electrochemical polarization, dc bias voltages between +700 mV and -500 mV were applied to the working electrode. The individual set voltages were not applied in a linear sequence but rather randomly (with repeating some of the set voltages) to check for the reversibility and reproducibility of the current-voltage characteristics and XPS features. At each set voltage the collection of XPS data was started after a steady state dc current was reached and surface sensitive Fe2p spectra were recorded with incident photon energy of 845 eV. During simultaneously performed electrochemical and XPS experiments the partial pressures of H_2 and H_2O were held constant at 0.25 mbar each, yielding a total pressure of 0.5 mbar in the chamber. Please note that in the given setup (both electrodes in the same atmosphere) a quantification of the produced amount of hydrogen is not feasible since hydrogen generated at one electrode is consumed at the other one. Thus the total composition of the atmosphere in the NAP-XPS chamber is not changed during electrochemical polarization of the sample.

2 Results of electrochemical measurements

2.1 DC measurements

In Fig. 2 of the main text the current-voltage characteristics of the LSF working electrode (i.e. a plot of the measured steady state dc current versus the applied overpotential) is shown. The overpotential η was calculated from the dc set voltages (U_{dc}) by subtracting the ohmic drop in the electrolyte and the contacting wires via

$$\eta = U_{dc} - R_{HF} \times I_{dc}. \quad (1)$$

Therein R_{HF} and I_{dc} denote the (ohmic) high frequency intercept measured by impedance spectroscopy (see below) and the dc current, respectively.

The current-overpotential curve in Fig. 2 is highly asymmetric with a strong non-linearity under cathodic polarization and a much weaker non-linearity in the anodic regime. Owing to this asymmetry, a fit to a function commonly applicable to electrode reactions in electrochemistry (e.g. based on exponential functions) was not successful over the entire voltage range with electrochemically meaningful parameters. To illustrate the unusual shape of the measured curve, we assumed charge transfer limited electrode kinetics with Butler-Volmer's (B-V) equation

$$I_{dc} = I_0 \cdot \left(e^{\frac{\alpha z e_0}{k_B T}\eta} - e^{\frac{(\alpha - 1) z e_0}{k_B T}\eta} \right) \quad (2)$$

describing the current-overpotential curve. Therein I_0 is the exchange current, α the symmetry factor (with α = 0.5 indicating a symmetric activation barrier of the rate limiting charge transfer step), and z the number of transferred charges; e_0, k_B, and T denote elementary charge, Boltzmann's constant, and absolute temperature, respectively. A comparison of the measured data and simulation results is shown in Fig. s1. The current values at strong anodic and strong cathodic polarization can only be described by using significantly different i_0 and z values in the B-V simulation. The part of the curve between 0 and -100 mV, however, cannot be explained by these functions at all. Only when assuming rather unrealistic parameters (1-α = 0.85, z = 2) this part of the I-η relationship can be simulated (but without explaining the data measured at high polarization). As a consequence, the steep increase of the current at cathodic polarizations beyond -20 mV is a strong indication of a fundamental change in the reaction mechanism at the electrode surface.

It should further be emphasized that the dc curve shown in Fig. 2 – apart from a small degradation directly after the first heating of the sample – could be measured reproducibly. (This is also true for the electrode's surface resistance measured by impedance spectroscopy.

Here, also only during the first couple of measurements some minor degradation of the surface resistance could be observed.) Irreversible changes of the electrode during the electrochemical experiment can thus be excluded as origin of the asymmetrically shaped curves.

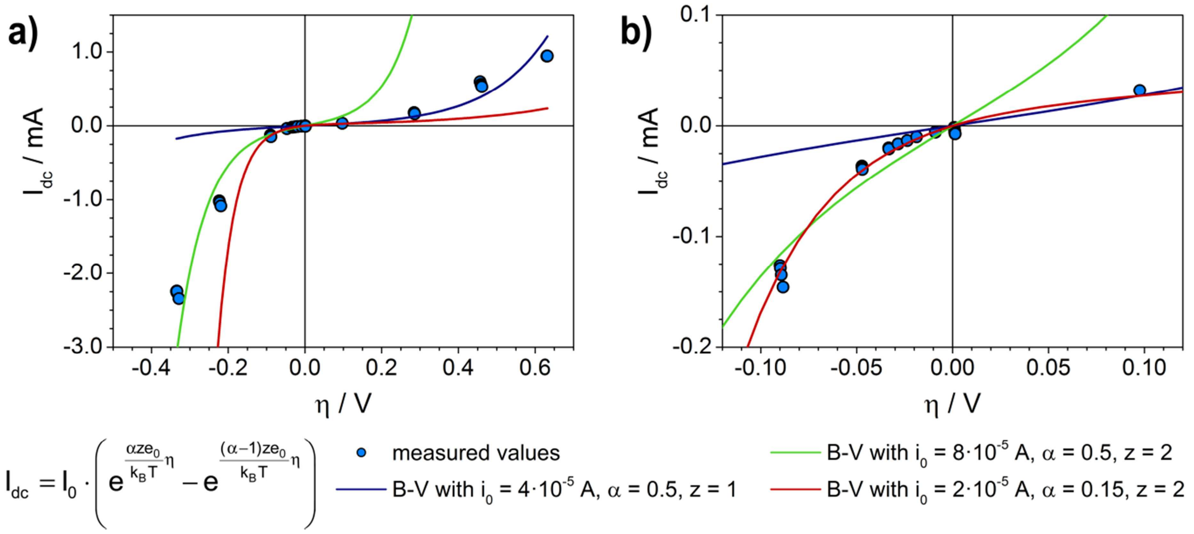

Figure s1: *Comparison of a measured current-overpotential curve with simulations based on Butler-Volmer's equation. a) Total voltage range. b) Magnification of moderate polarization from (a). It becomes obvious that the entire curve cannot be explained by means of classical Butler-Volmer kinetics.*

2.2 Impedance spectroscopy

Typical impedance spectra under equilibrium and upon polarization are depicted in Fig. s2. Qualitatively, each of the spectra consists of the same features: (i) a Z_{re}-axis intercept, (ii) a relatively well separated (semicircle like) shoulder in the high frequency regime, (iii) an additional shoulder in the medium frequency range and (iv) a slightly depressed low frequency arc. The high frequency axis (R_{HF}) intercept can mainly be assigned to the resistance of ion conduction in YSZ [7] and a ca. 5 Ω resistance of the measurement wires and contacts. The electrode polarization (dc measurements) was corrected for the ohmic losses caused by this high frequency offset resistance (R_{HF}) – see above. The very small high frequency semicircle like shoulder is most probably caused by an ion transfer resistance at the electrode|electrolyte interface and an according interfacial capacitance[7] and partly also the counter electrode may contribute to this impedance feature. In agreement with a similar study on $SrTi_{0.7}Fe_{0.3}O_{3-\delta}$ electrodes[8] the medium frequency shoulder and the low frequency arc are

attributed to in-plane electron transport within the MIEC thin film and the electrochemical reaction at the MIEC surface, respectively. The capacitance involved is the chemical capacitance of the mixed conducting electrode material LSF.[9] In all cases considered here the low frequency arc and thus the surface reaction resistance dominates the polarization resistance of the LSF electrode. Therefore we can conclude that the polarization of the working electrode is virtually homogeneous for the geometry used in this study. In case of a homogeneously polarized mixed conducting electrode with surface limited kinetics the overpotential η directly translates into a change of the oxygen chemical potential within the bulk of the electrode $\mu(O_2)_{MIEC}$ and $\Delta\mu(O_2)_{surface} = \mu(O_2)_{MIEC} - \mu(O_2)_{gas} = 4e_0\eta$ holds. Thus, the thermodynamic driving force for the surface reaction $\Delta\mu(O_2)_{surface}$ can be easily related to the applied overpotential.

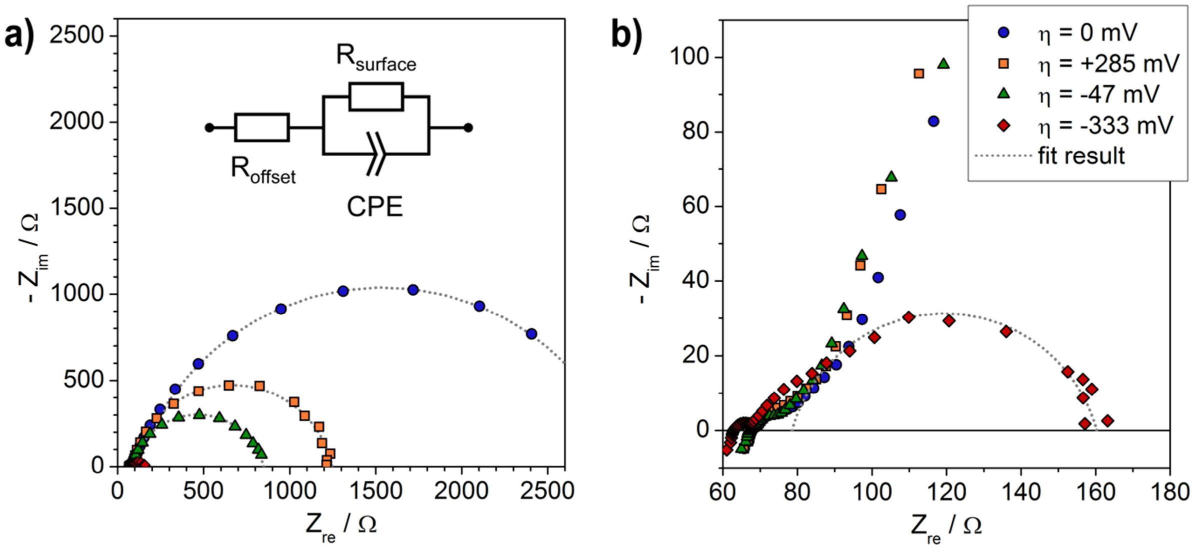

Figure s2: *a) Impedance spectra (Nyquist plot) measured at 620 ±15°C in 0.25 mbar* H_2 *+ 0.25 mbar* H_2O *atmosphere under equilibrium conditions (circles), as well as under anodic (squares) and cathodic (triangles and diamonds) polarization. The dotted curves show the results obtained by fitting to the equivalent circuit depicted in the inset. b) Magnification of the spectrum measured at a cathodic overpotential of -333 mV from (a).*

As shown in Fig. s2 the low frequency arc is decreased by any dc bias. This bias dependence of the differential impedance fits well to non-linear current-voltage characteristics and is a common behavior of electrode reactions. Uncommon, however, is the very strong bias dependence already found for rather small cathodic bias values, see -47 mV in Fig. s2. For

evaluation of the resistance of the electrochemical reaction at the electrode surface, the low frequency part of the spectra (i.e. only the low frequency arc) was fitted to the equivalent circuit shown in the inset of Fig. s2a. In this circuit the oxygen exchange reaction at the LSF surface and the chemical capacitance of LSF are represented by the resistor $R_{surface}$ and the constant phase element[10] CPE, respectively. The serial resistor R_{offset} accounts for all resistive contributions with higher relaxation frequencies and is thus larger than R_{HF}. Even though this equivalent circuit strongly simplifies the impedance response of the investigated electrodes, a reasonable estimate of the dominating surface-related resistance $R_{surface}$ is possible for homogeneous polarization.[8]

3 XPS results

3.1 Fe2p and valence band spectra

Fe2p photoelectron spectra measured with 845 eV incident photon energy and at different electrochemical polarization are shown in the insets in Fig. 2 of the main text and details are given in Fig. s3a. The information depth in these NAP-XPS experiments is 0.5 ± 0.1 nm (this value is obtained by calculation of the inelastic mean free path of photoelectrons in LSF according to the NIST SR 71 database using TPP-2M equation). Depending on the applied electrochemical overpotential, two fundamentally different types of Fe2p spectra can be found in Fig. s3a:

(i) Between about -20 mV and the highest anodic overpotential two pronounced peaks at ~710 eV and ~724 eV binding energy (BE) were observed, which can be attributed to $Fe2p_{3/2}$ and $Fe2p_{1/2}$, respectively. Both binding energy values are in reasonable agreement with literature values of Fe^{+II} and/or Fe^{+III}.[11] A $Fe2p_{3/2}$ satellite at ~716 eV is weakly visible in the spectrum measured without electrochemical polarization and corresponds well to a Fe^{+II} species. This suggests a significant amount of Fe^{+II} at the surface, while bulk-sensitive studies on $La_{0.6}Sr_{0.4}FeO_{3-\delta}$[12] indicate mostly Fe^{+III} to be present. A high reducibility of oxide surfaces was already observed for ceria-based anodes[13] and highlights the importance of in-situ investigations.[14] (The binding energy of an additional feature at ~730 eV principally accords to the satellite of $Fe2p_{1/2}$. However, owing to its high intensity and relatively sharp peak shape additional contributions – e.g. of Auger lines – cannot be excluded.)

(ii) In addition to the peaks described above, at cathodic overpotentials more negative than -20 mV two further peaks at about 707 eV and 720 eV were observed. These peaks can be related to $Fe2p_{3/2}$ and $Fe2p_{1/2}$ of metallic iron, respectively.[11] It should be mentioned that by

variation of the measurement position beam damage of the LSF was excluded to be the origin of the described chemical changes during XPS experiments.

XPS valence band spectra of LSF were recorded with a photon energy of 140 eV (the photon energy was select in that way, that the kinetic energy of the emitted photoelectrons is the same as in case of Fe2p spectra and therefore the information depth is identical) and at the same electrochemical polarization as the Fe2p spectra – see Fig. s3b. Interestingly, for cathodic polarization leading to metallic iron peaks in the Fe2p spectra also a Fermi edge appeared in the very low binding energy range (see arrows in Fig. s3b). Correlation of these two changes in the XPS spectra can be regarded as a strong evidence for the near-surface formation of a metallic Fe phase. It should be emphasized that the evolution of a metallic Fe species at the electrode surface did not correspond to an irreversible decomposition of the LSF electrode. Rather, a (surprisingly) reversible behavior was found in electrochemical and XPS experiments (cf. Figs. 2 and 3a of the main text).

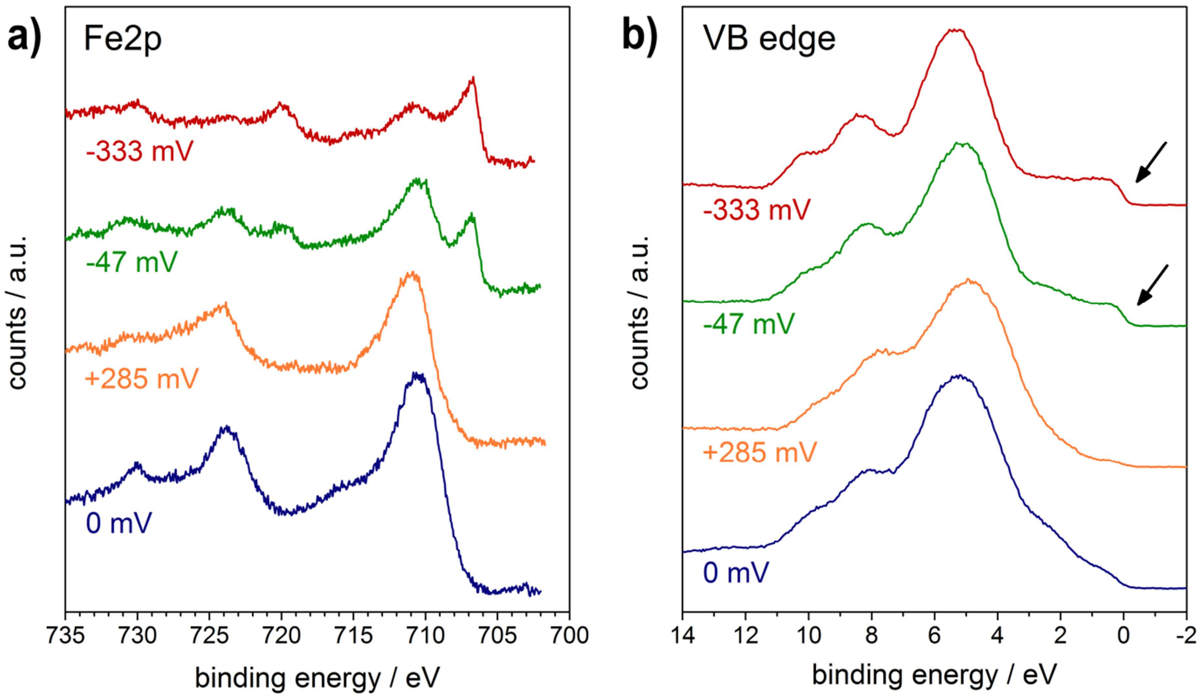

Figure s3: *a) Fe2p XPS spectra measured with 845 eV photon energy; each spectrum is plotted with the same scale. The formation of metallic Fe and a strong decrease of the total Fe signal are clearly visible under cathodic polarization. The applied overpotentials are indicated for each curve. b) Valence band spectra measured with 140 eV photon energy; each spectrum is plotted with the same scale. The applied overpotential is given for each curve. The Fermi edges appearing under cathodic polarizations are indicated by arrows.*

3.2 Sr3d, La3d and O1s spectra

Besides Fe2p spectra also La3d, Sr3d and O1s spectra were recorded at each electrochemical polarization state – they are depicted in Fig. s4. However, only minor changes occurred in these XPS spectra, which were by far not as pronounced as in case of Fe2p and valence band edge spectra. Apart from some minor shifts in binding energy (which may be due to insufficient BE correction owing to a missing Fermi edge) no significant changes in the La3d, Sr3d and O1s spectra were found. This observation further supports the assumption that the metallic iron formed under cathodic polarization is not part of the perovskite phase. Otherwise much more pronounced changes in the La3d, Sr3d and O1s spectra are to be expected.

Near-surface Sr3d spectra measured on $(La,Sr)CoO_{3-\delta}$ (LSC) electrodes in oxygen atmospheres under electrochemical polarization were reported in Ref. [15]. In the cited study two Sr species – one at lower BE and one at higher BE – were observed and interpreted as lattice and surface species, respectively. Similarly, in our reducing atmosphere the shape of the spectra may be explained by contribution of two Sr species (cf. Fig. s4c). However, a quantitative analysis of the spectra (except Fe) is beyond the scope of the present paper.

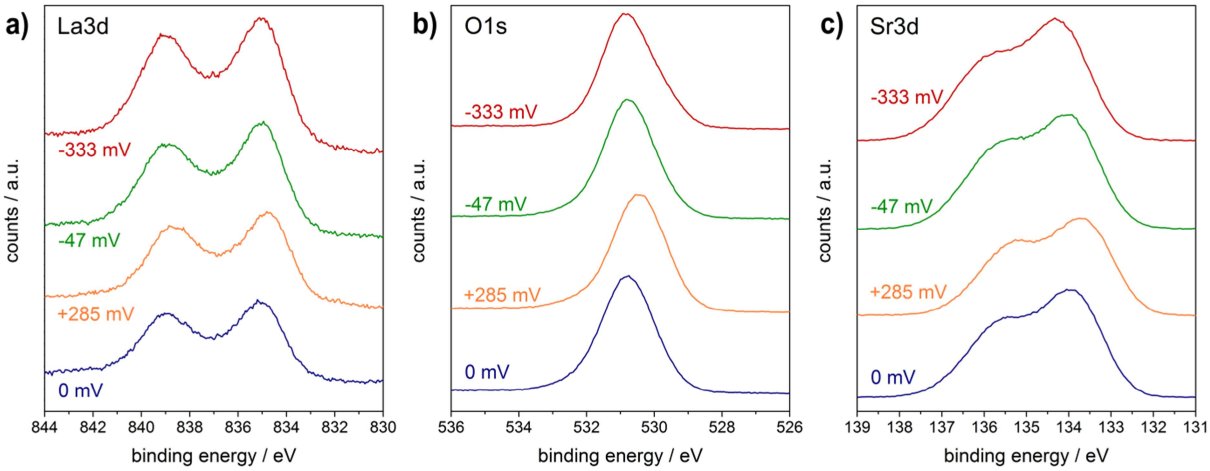

***Figure s4**: a) La3d XPS spectra measured with 975 eV photon energy; each spectrum is plotted with the same scale. The applied overpotentials are depicted at the respective curve. b) O1s XPS spectra measured with 650 eV photon energy; each spectrum is plotted with the same scale and applied overpotentials are depicted at the respective curve. c) Sr3d XPS spectra measured with 252 eV photon energy; each spectrum is plotted with the same scale and applied overpotentials are depicted at the respective curve.*

3.3 Fitting of Fe2p XPS data

Fe2p spectra were deconvoluted (software: CasaXPS) using a simplified peak model with the main objective to quantify the relative proportions of metallic and oxidic iron. For this means, only the $Fe2p_{3/2}$ peak was considered in the fit. A line-shape related to the asymmetric Lorentian was used for the oxidic and metallic contribution – commonly referred to as LF line-shape[16]. This line-shape has an exponential decay at the high-binding energy tail of the synthetic component, so the synthetic peak intensity (nearly) meets the background at the end of the fitting region. Peak parameters LF(1.3,2,10,0) and LF(0.8,2,15,0) were used for the $Fe^{+II/+III}$ and Fe^{0} peaks, respectively. The authors are aware that this method of peak fitting is a strong simplification of the actually very complex Fe peak structure.[17, 18] However, this model is – due to the low number of fitting parameters and constraints – very reliable to separate the peak contributions of metallic and oxidic iron.

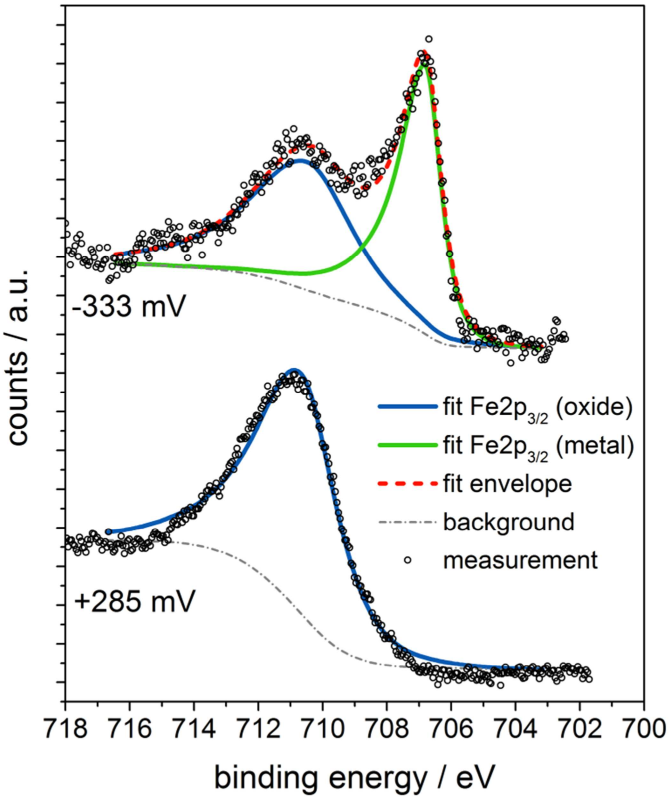

Figure s5: *Fit of the $Fe2p_{3/2}$ spectra at overpotentials of -333 mV and +285 mV; the intensity scale of the two spectra was adjusted*

A fit of the $Fe2p_{3/2}$ spectra measured at -333 mV and +285 mV is shown in Fig. s5, illustrating the pronounced differences between surfaces of anodically and cathodically polarized LSF. In this fit we considered a metallic (Fe^{0}) and one oxidized iron species (Fe^{+II} and/or Fe^{+III}). The resulting binding energies of the $Fe2p_{3/2}$ peak for the oxidic and the metallic Fe species are

710.3 ± 0.2 eV and 706.8 ± 0.1 eV, respectively. These results are in good agreement with literature data.[11] The quantification of the relative amounts of +II and +III species, however, was not unambiguously possible. This is at least partly owing to the energy calibration of XP spectra, which was only possible with respect to the valence band edge. (Please note: the error caused by the uncertainty of the X-ray monochromator is about ±0.15 eV.)

References:

[1] Innovative Station for In Situ Spectroscopy, https://www.helmholtz-berlin.de/pubbin/igama_output?modus=einzel&sprache=en&gid=1607&typoid=50740 , accessed 2014, Sept. 24th.

[2] H. Bluhm, M. Hävecker, A. Knop-Gericke, E. Kleimenov, R. Schlögl, D. Teschner, V. I. Bukhtiyarov, D. F. Ogletree, M. Salmeron, *The Journal of Physical Chemistry B* **2004**, *108*, 14340-14347.

[3] A. Knop-Gericke, E. Kleimenov, M. Hävecker, R. Blume, D. Teschner, S. Zafeiratos, R. Schlögl, V. I. Bukhtiyarov, V. V. Kaichev, I. P. Prosvirin, A. I. Nizovskii, H. Bluhm, A. Barinov, P. Dudin, M. Kiskinova, in *Advances in Catalysis, Vol. Volume 52* (Eds.: C. G. Bruce, K. Helmut), Academic Press, **2009**, pp. 213-272.

[4] ISISS Station, https://www.helmholtz-berlin.de/pubbin/igama_output?modus=einzel&sprache=en&gid=1671, accessed 2014 Sept. 24th.

[5] A. K. Opitz, J. Fleig, *Solid State Ionics* **2010**, *181*, 684–693.

[6] A. K. Opitz, A. Lutz, M. Kubicek, F. Kubel, H. Hutter, J. Fleig, *Electrochimica Acta* **2011**, *56*, 9727– 9740.

[7] F. S. Baumann, J. Fleig, H. U. Habermeier, J. Maier, *Solid State Ionics* **2006**, *177*, 1071-1081.

[8] A. Nenning, A. K. Opitz, T. Huber, J. Fleig, *Phys. Chem. Chem. Phys.* **in press**.

[9] J. Jamnik, J. Maier, *Physical Chemistry Chemical Physics* **2001**, *3*, 1668-1678.

[10] J. Fleig, *Solid State Ionics* **2002**, *150*, 181-193.

[11] M. Descostes, F. Mercier, N. Thromat, C. Beaucaire, M. Gautier-Soyer, *Applied Surface Science* **2000**, *165*, 288-302.

[12] M. Kuhn, S. Hashimoto, K. Sato, K. Yashiro, J. Mizusaki, *Solid State Ionics* **2011**, *195*, 7-15.

[13] W. C. Chueh, A. H. McDaniel, M. E. Grass, Y. Hao, N. Jabeen, Z. Liu, S. M. Haile, K. F. McCarty, H. Bluhm, F. El Gabaly, *Chemistry of Materials* **2012**, *24*, 1876-1882.

[14] C. Rameshan, W. Stadlmayr, C. Weilach, S. Penner, H. Lorenz, M. Hävecker, R. Blume, T. Rocha, D. Teschner, A. Knop-Gericke, R. Schlögl, N. Memmel, D. Zemlyanov, G. Rupprechter, B. Klötzer, *Angewandte Chemie International Edition* **2010**, *49*, 3224-3227.

[15] E. J. Crumlin, E. Mutoro, W. T. Hong, M. D. Biegalski, H. M. Christen, Z. Liu, H. Bluhm, Y. Shao-Horn, *The Journal of Physical Chemistry C* **2013**, *117*, 16087-16094.

[16] Lorentzian Asymmetric Lineshape, http://www.casaxps.com/help_manual/manual_updates/LA_Lineshape.pdf, accessed 2014, July, 2nd.

[17] M. Aronniemi, J. Sainio, J. Lahtinen, *Surf Sci* **2005**, *578*, 108-123.

[18] M. C. Biesinger, B. P. Payne, A. P. Grosvenor, L. W. M. Lau, A. R. Gerson, R. S. C. Smart, *Applied Surface Science* **2011**, *257*, 2717-2730.

References abbreviated in the main text:

C. Rameshan, W. Stadlmayr, C. Weilach, S. Penner, H. Lorenz, M. Hävecker, R. Blume, T. Rocha, D. Teschner, A. Knop-Gericke, R. Schlögl, N. Memmel, D. Zemlyanov, G. Rupprechter, B. Klötzer, *Angew. Chem.* **2010**, *122*, 3292-3296; *Angew. Chem. Int. Ed.* **2010**, *49*, 3224-3227.

C. Zhang, Y. Yu, M. E. Grass, C. Dejoie, W. Ding, K. Gaskell, N. Jabeen, Y. P. Hong, A. Shavorskiy, H. Bluhm, W.-X. Li, G. S. Jackson, Z. Hussain, Z. Liu, B. W. Eichhorn, *J. Am. Chem. Soc.* **2013**, *135*, 11572-11579.

Beschleunigung der elektrochemischen Wasserspaltungskinetik durch polarisationsgetriebene Bildung von oberflächennahem Eisen(0): Eine in-situ XPS Studie an Perowskit-Elektroden**

Alexander K. Opitz, Andreas Nenning, Christoph Rameshan, Raffael Rameshan, Raoul Blume, Michael Hävecker, Axel Knop-Gericke, Günther Rupprechter, Jürgen Fleig und Bernhard Klötzer*

**Diese Arbeit wurde vom österreichischen Fonds zur Förderung der wissenschaftlichen Forschung (FWF) im Rahmen der Projekte F4502/03/09 (SFB FOXSI) und W1243 finanziell unterstützt. Wir bedanken uns beim Helmholtz-Zentrum Berlin für die Zurverfügungstellung von Strahlzeit am Strahlrohr ISIS-PGM des Synchrotron BESSY II und beim den BESSY-Mitarbeitern für die Unterstützung. Darüber hinaus wurde diese Arbeit durch das Seventh Framework Programme (FP7/2007-2013) der Europäischen Union unterstützt (Grant Agreement Number 226716). Bei Dr. Dmitry Zemlyanov möchten wir uns für hilfreiche Diskussionen bedanken.

Kurzfassung:
Auf der Suche nach optimierten Kathodenmaterialien für die Hochtemperaturelektrolyse gelten gemischt leitende Oxide als sehr vielversprechende Kandidaten. In dieser Studie berichten wir über grundlegend neue Einblicke in das Zusammenspiel zwischen der Oberflächenchemie und der elektrokatalytischen Aktivität von Lanthan-Ferrit-basierten Kathoden für die Hochtemperaturelektrolyse. Dazu wurden simultan Messungen mittels („near ambient pressure") Röntgen-Fotoelektronenspektroskopie (NAP-XPS) und Impedanzspektroskopie auf elektrochemisch polarisierten $La_{0.6}Sr_{0.4}FeO_{3-\delta}$ (LSF) Dünnschicht- Elektroden durchgeführt. Unter kathodischer Polarisation wurde die Bildung von Fe^0 auf der LSF Oberfläche beobachtet, was gleichzeitig eine deutliche Verbesserung der elektrochemischen Wasserspaltungsaktivität der Elektroden zur Folge hatte. Diese Korrelation deutet auf einen grundlegend anderen Wasserspaltungsmechanismus in Anwesenheit von metallischem Eisen hin, was neue Wege bei der Suche nach Elektroden mit erhöhter Wasserspaltungsaktivität aufzeigen kann.

Elektrochemische Festoxid-Zellen sind sehr vielversprechende Aggregate für die Umwandlung chemischer in elektrische Energie (Festoxid-Brennstoffzellen, SOFCs) als auch für den umgekehrten Prozess (Festoxid-Elektrolysezellen, SOECs). SOFCs werden bereits kommerziell genutzt, ein Keramik-Metall-Verbundwerkstoff (Cermet) aus Ni und Yttrium-stabilisiertem Zirkoniumoxid (YSZ) ist bereits erfolgreich als Anodenmaterial in Verwendung.[1] Im Gegensatz dazu ist die Entwicklung von SOECs deutlich weniger weit fortgeschritten. Oftmals werden SOFCs einfach im "Umkehrmodus" zur Elektrolyse verwendet, was eine starke Degradation der Ni/YSZ Elektrode zur Folge haben kann.[2,3] Eine mögliche Lösung wäre der Einsatz von gemischt ionisch und elektronischen Leitern (MIEC) anstelle von Ni/YSZ. MIECs werden bereits in oxidierender Atmosphäre als Elektroden verwendet und gemischt leitende Oxide wie z.B. $La_{0.6}Sr_{0.4}FeO_{3-\delta}$ (LSF) sind ebenfalls interessante Kandidaten für zukünftige SOEC Kathoden, da - im Gegensatz zur viel kleineren Drei-Phasengrenze im Falle von Ni/YSZ - größere Teile der Elektrodenoberfläche elektrochemisch aktiv werden können.[4-10] Allerdings ist wenig über die Oberflächenchemie von gemischt leitenden perowskitartigen Elektroden in reduzierender Atmosphäre und ihrer Auswirkung auf die elektrochemischen Eigenschaften dieser Materialien bekannt. Insbesondere Kenntnisse über Änderungen in der oberflächen(nahen) Zusammensetzung und der Kationenvalenzzustände unter elektrochemischer Polarisation fehlen fast gänzlich.

Umgebungsdrucknahe Röntgen-Fotoelektronenspektroskopie (NAP-XPS) ist eine leistungsfähige Methode zur Untersuchung von MIECs unter elektrochemischer Polarisation, die Informationen über adsorbierte Spezies als auch über den Zustand oberflächennaher Kationen einer polarisierten Elektrode liefert.[11-13] In situ NAP-XPS Studien wurden bereits auf Perowskit-Elektroden in oxidierender Atmosphäre[14-16] und auf Ceroxid-basierten Elektroden unter reduzierenden Bedingungen durchgeführt.[17-19] Jedoch gelang es bisher nicht, gleichzeitig die elektrochemische Oberflächenaktivität und die Oberflächenzusammensetzung einer gemischtleitenden Perowskit-Elektrode in einer H_2/H_2O Atmosphäre unter Einstellung eines klar definierten elektrochemischen Polarisationszustandes zu bestimmen. Vor allem eine wohl-definierte und homogene Polarisation der LSF Elektrode (die ja unter reduzierenden Bedingungen ein schlechter Elektronenleiter ist) ist die Voraussetzung, um einen direkten Zusammenhang zwischen Änderungen in der elektrochemische Oberflächenaktivität und den beobachteten Veränderungen in der Oberflächenchemie abzuleiten.

Im Folgenden wird ausgeführt, wie synchrotronbasierte NAP-XPS und Impedanz-spektroskopie Messungen simultan durchgeführt wurden, um so Änderungen in der

Oberflächenchemie und in der Wasserspaltungskinetik des perowskitartigen Gemischtleiters $La_{0.6}Sr_{0.4}FeO_{3-\delta}$ (LSF) unter elektrochemischer Polarisation in H_2/H_2O Atmosphäre zu untersuchen. Dabei stellt die geringe elektronische Leitfähigkeit von LSF unter reduzierenden Bedingungen ein Problem dar.[20, 21] Dies kann jedoch durch einen dünnen, mikrostrukturierten, metallischen Film kompensiert werden, der durch seine Funktion als Stromsammler eine nahezu homogene Polarisation der Arbeitselektrode ermöglicht[8, 22] und damit eine homogene Triebkraft der elektrochemischen Oberflächenreaktion gewährleistet (siehe auch Supporting Informations). Diese Stromsammler wurden als ineinandergreifende Pt-Finger ausgeführt, die sich unter der 200 nm dicken LSF Schicht befinden. Die derartig aufgebauten Modellkomposit-Arbeitselektroden wurden auf einkristallinem YSZ - auf dessen Rückseite eine poröse Gegenelektrode aufgebracht war - abgeschieden (Abbildung 1). Einzelheiten zur Herstellung von LSF Arbeitselektroden mit eingebetteten Stromabnehmern und der porösen Gegenelektroden werden in den Supporting Informations ausgeführt.

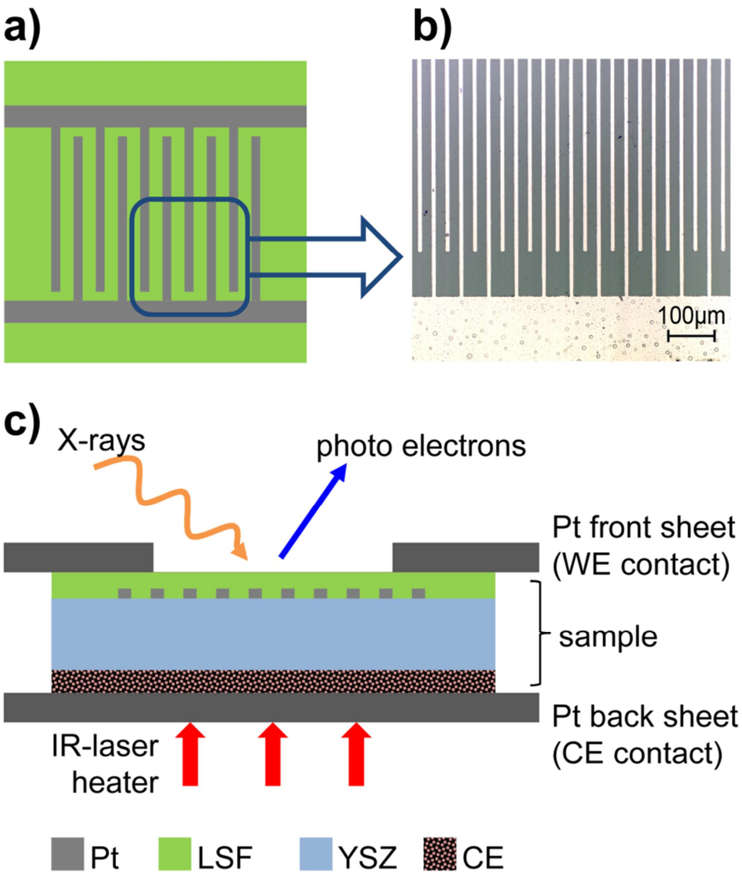

Abbildung 1. *a) Skizze und b) lichtmikroskopische Aufnahme einer Probe mit einer Dünn-Film LSF Elektrode und darunterliegenden Stromabnehmern. c) Skizze (Querschnitt) einer Probe für NAP-XPS-Messungen (WE-Arbeitselektrode, CE-Gegenelektrode).*

Die NAP-XPS-Messungen wurden am ISISS Strahlrohr des HZB/BESSY II Synchrotron in Berlin durchgeführt.[23] NAP-XPS Experimente unter elektrochemischer Polarisation wurden durch das Anbringen der Proben zwischen zwei dünnen Platinblechen realisiert (Deckblech mit einem Loch), welche als mechanische Fixierung und als elektrische Kontakte der beiden Elektroden fungierten (Abbildung 1c). Dieser neuartige Aufbau erlaubt eine gleichzeitige Messung der elektrochemischen Reaktionsgeschwindigkeit und der Oberflächenaktivität (durch elektrochemische Gleichstrommessung und Impedanzspektroskopie) sowie der chemischen Zusammensetzung der Oberfläche und der Valenzzustände von oberflächennahen Kationen (mittels XPS; für experimentelle Details siehe Supporting Informations).

Die gemessene Strom-Überspannungs-Kennlinie einer LSF Arbeitselektrode ist in Abbildung 2 dargestellt. Drei ausgewählte Fe2p Spektren, die drei unterschiedlichen Polarisationen entsprechen, werden ebenso dargestellt und zeigen, dass sich eine metallische Eisen-Spezies bereits bei relativ niedrigen kathodischen Überspannungen ausbildet. Gleichzeitig mit der Bildung von Fe^0 wird die elektrochemische Wasserspaltungsaktivität der LSF Oberfläche stark erhöht, was zu einer stark asymmetrischen Strom-Spannungs-Kennlinie führt, welche nicht mit einer exponentiellen Funktion wie zum Beispiel der Butler-Volmer-Gleichung mit elektrochemisch sinnvollen Parametern erklärt werden kann (Supporting Informations, Abbildung S1). Dies deutet auf einen mechanistischen Wechsel der Reaktionskinetik von $H_2O + 2e^- \rightleftharpoons H_2 + O^{2-}$ an der LSF Oberfläche hin. Das kathodisch gebildete Fe^0 wird nach Abschalten der Polarisation schnell re-oxidiert (in der Zeit zwischen zwei simultanen XPS/Impedanz Messungen, etwa 200 bis 600 s). Dementsprechend ist die Strom-Spannungs-Kurve in Abbildung 2 reversibel. Infolge dieser Reversibilität ist eine Beobachtung der kathodisch gebildeten metallischen Fe-Spezies offensichtlich nur mittels in-situ Experimenten möglich, wie sie auch in der vorliegenden Studie angewandt wurden.

An jedem Punkt der Strom-Spannungs-Kennlinie (d.h., für unterschiedliche Reaktionsraten) wurden simultan NAP-XPS und Impedanzmessungen durchgeführt. Die wichtigsten Ergebnisse der Impedanzmessungen sind (zu Einzelheiten der Datenanalyse siehe Supporting Informations): Aus den aufgenommenen Impedanzspektren (vergleiche Supporting Informations, Abbildung S2) wurde ein Oberflächenwiderstand ($R_{surface}$) bestimmt. Der reziproke, flächenbezogene Oberflächenwiderstand $1/(R_{surface}A)$, der ein Maß für die elektrochemische Aktivität der LSF Oberfläche für die Wasserspaltungs-/Wasserstoffoxidationsreaktion darstellt, ist in Abbildung 3a gegen die Überspannung η aufgetragen. In dieser Graphik wird die starke Asymmetrie der Elektrodenkinetik noch

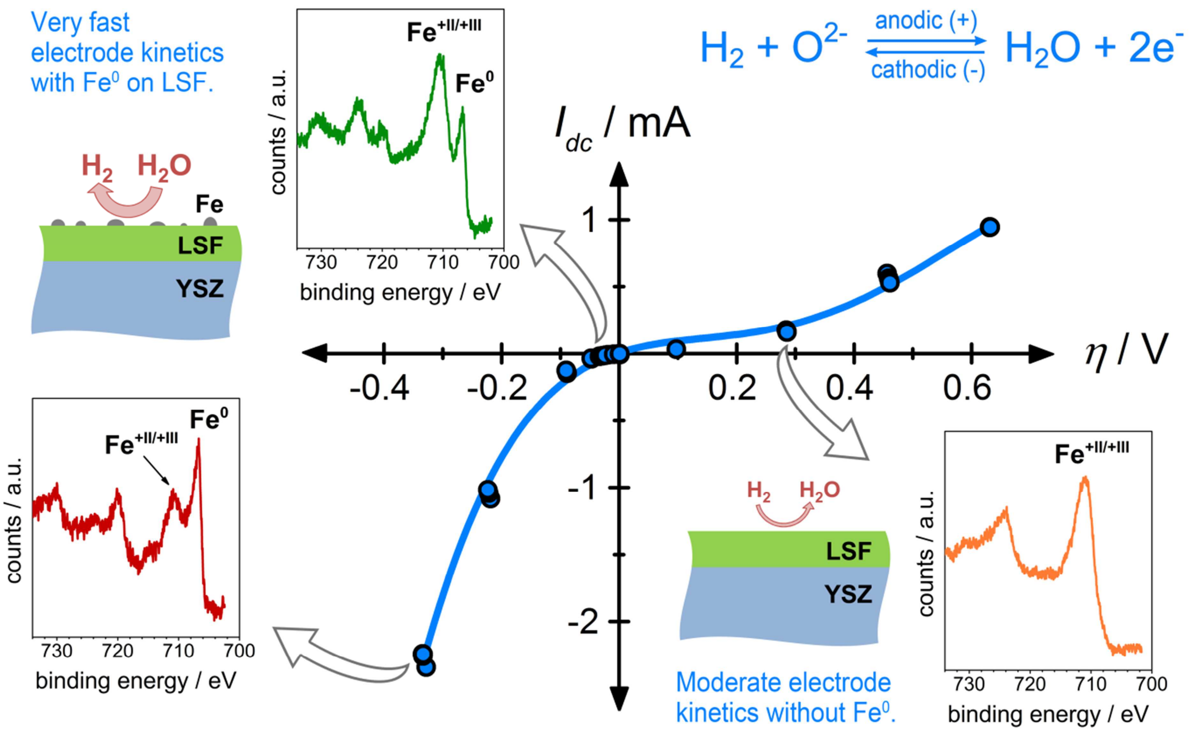

Abbildung 2. *Strom-Überspannungs-Kennlinie (I_{dc} vs. η) von LSF in einer feuchten reduzierenden Atmosphäre (0,25 mbar H_2 + 0,25 mbar H_2O). Die Kreise stehen für Messwerte; die Linie ist kein Fit, sondern stellt eine Hilfslinie dar. Oben rechts ist die Reaktion, die auf der Oberfläche der LSF Arbeitselektrode abläuft, gegeben. Für ausgewählte Punkte der Kurve (durch Pfeile markiert) sind Fe2p XPS-Spektren als Inset gezeigt. Die Skizzen zeigen jeweils den Status der LSF-Oberfläche und die daraus resultierende Reaktivität.*

deutlicher sichtbar (insbesondere bei kleinen Überspannungen): Eine starke anodische Polarisation (η = +285 mV) verursacht eine Zunahme von $1/(R_{surface}A)$ um einen Faktor von etwa drei bis vier, wohingegen bereits eine kleine kathodische Polarisation (η = –47 mV) zu etwa der gleichen Steigerung führt. Eine Erhöhung der kathodischen Überspannung um 70 mV (von -20 auf -90 mV) verbessert die Oberflächenaktivität, die durch $1/(R_{surface}A)$ quantifiziert, um etwa eine Größenordnung. In Abbildung 3a wird diese starke Asymmetrie durch eine nahezu stufenförmige Nichtlinearität der Wasserspaltungsaktivität im kathodischen Bereich reflektiert und findet sein Pendant in den im Folgenden beschriebenen XPS-Ergebnissen.

Die gemessenen Fe2p XPS-Spektren wurden mit einem vereinfachten Modell analysiert, da es das Hauptziel war, die relativen Anteile von metallischem und oxidischem Eisen zu

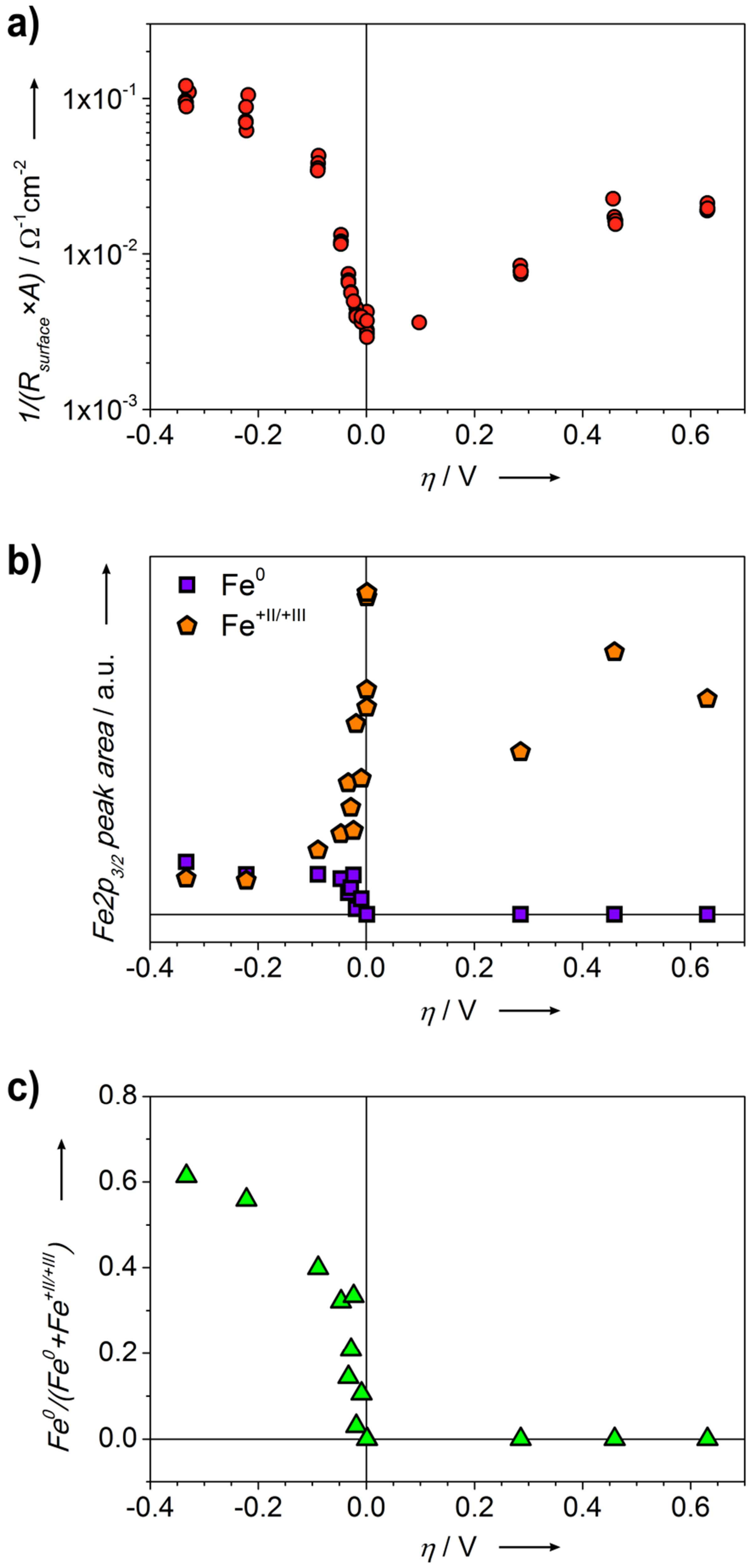

Abbildung 3. *a) Flächenbezogener, reziproker Oberflächenwiderstand (gemessen mit Impedanzspektroskopie) gegen Überspannung η. b) Fe2p$_{3/2}$ Peakflächen (erhalten aus XPS-Spektren) der unterschiedlichen Fe-Spezies als Funktion von η. c) Anteil von Fe^{0} berechnet aus den Werten der Peakflächen in (b), aufgetragen gegen die Überspannung.*

quantifizieren. Somit wurden nur die $Fe2p_{3/2}$ Signale für den Fit herangezogen (für Details siehe Supporting Informations, Abschnitt 3.3). Die resultierenden Peak-Flächen beider Fe-Spezies (metallisch und oxidisch) und der Anteil von Fe^0 (in Bezug auf die gesamte Eisenmenge) sind jeweils gegen die Überspannung η in Abbildung 3b bzw. c aufgetragen. Die Menge von Fe^0 und der relative Anteil von metallischem Eisen an der Gesamteisenmenge erhöhen sich abrupt für kathodische Polarisationen von mehr als ca. -20 mV. Im gleichen Spannungsbereich erhöht sich die Wasserspaltungsaktivität der LSF Elektrode (gegeben durch $1/(R_{surface}A)$) ebenfalls stark. Diese Korrelation deutet auf einen grundlegenden Unterschied im Wasserspaltungsmechanismus auf LSF in Gegenwart und Abwesenheit von Fe^0 hin.

Anhand der vorliegenden Daten kann die genaue strukturelle Morphologie der metallischen Eisenspezies noch nicht identifiziert werden. Oberflächennahes Eisen könnte zu Fe^0 reduziert werden, während es immer noch ein Teil des Perowskitgitters ist, jedoch erscheint die Bildung von metallischen Eisenpartikeln auf der LSF-Oberfläche wahrscheinlicher. Die Ausbildung einer Fermi-Kante in den Valenzband Spektren (Supporting Informations, Abbildung S3b) deutet stark auf die Bildung einer metallische Phase hin, und legt somit den letzteren Mechanismus nahe; dies bedeutet eine elektrochemisch angetriebene Ausbildung von metallischen Fe-Partikeln auf der LSF-Oberfläche, wie in Abbildung 2 skizziert. Dies wird zusätzlich durch die Beobachtung gestützt, dass sich die Fe-Peakfläche (oxidisches und metallisches) bei der Bildung von Fe^0 stark verringert (Abbildung 3b; Supporting Informations, Abbildung S3a). Unter der Annahme, dass die Fe-Partikel deutlich größer sind als die mittlere freie Weglänge der Fotoelektronen (hier ca. 0,5 nm), würde nach Fe^0-Partikelbildung eine signifikante Menge des oberflächennahen Eisens unzugänglich für XPS-Messungen werden. Die Bildung von metallischen Eisenteilchen bei bereits kleiner kathodischer Polarisation ist auch in Übereinstimmung mit der Tatsache, dass unter diesen experimentellen Bedingungen das LSF relativ nahe an seiner thermodynamischen Stabilitätsgrenze ist.[21] Darüber hinaus wurde kürzlich die chemisch getriebene Bildung von Metallpartikeln aus perowskitartigen SOFC Anodenmaterialien unter reduzierenden Bedingungen, sowie der vollständig reversible Wiedereinbau dieser Teilchen unter oxidierenden Bedingungen, berichtet.[24, 25] Eine ähnliche chemisch getriebenen Bildung von metallischem Pd-Teilchen auf Pd-dotierten $LaFeO_3$ Katalysatoren wurde unter reduzierenden Bedingungen ebenfalls gefunden.[26] Ein Grund für die beobachtete Stabilität der LSF Elektroden unter Reduktionsbedingungen könnte die oberflächennahe Bildung von Ruddlesden-Popper ähnlichen Phasen bei Fe^0-Partikelevolution sein.[24] Hohe chemische

Stabilität solcher Phasen in H_2-Atmosphäre wurde für Cr/Ni-hältige Ruddlesden-Popper Oxide gezeigt.[27]

Die stark verbesserte elektrochemische Kinetik - sogar für kleine kathodische Überspannungen - könnte durch das Vorhandensein von Fe^0-Partikel an der Oberfläche ausgelöst werden, welche die Kinetik der Wasserspaltungsreaktion beschleunigen. Aber auch die restliche Fe-verarmte Oxidoberfläche (unbekannter Kristallstruktur) könnte für die deutlich schnellere Elektrodenkinetik verantwortlich sein; etwa durch eine drastische Veränderung des Fe^{+II}/Fe^{+III} Verhältnisses an der Oxidoberfläche. Darüber hinaus ist ein komplexes mechanistisches Zusammenspiel von sowohl Metall- als auch Oxid-Phase denkbar; Zweiphasen-promotierte elektrochemische Kinetik wurde für Perowskit-Elektroden in Sauerstoff beschrieben.[14, 28, 29]

Nur durch weitere Messungen können Details zur Fe^0-hältigen Phase erhalten werden, sowie die eher komplizierten mechanistischen Gründe für die ungewöhnliche I-V-Kurve aufgeklärt und des Weiteren Möglichkeiten gefunden werden, solche hochaktive SOEC-Kathoden auch durch chemische Präparation anstatt durch Polarisation herzustellen. In jedem Fall unterstreicht die gemessene sprunghafte Verbesserung der Elektrodenperformance durch Fe^0-Segregation die hohe Komplexität eines solchen Systems, bietet jedoch auch neue Möglichkeiten bei der Suche nach neuartigen oxidbasierten Elektroden für die elektrochemische Wasserspaltung bei hohen Temperaturen.

Referenzen:

[1] N. Q. Minh, Solid State Ionics 2004, 174, 271 – 277.

[2] J. E. OBrien, J. J. Hartvigsen, S. Elangovan, C. M. Stoots, J. S. Herring, P. A. Lessing, J. Fuel Cell Sci. Tech. **2005**, *2*, 156 – 163.

[3] J. S. Herring, J. E. OBrien, C. M. Stoots, G. L. Hawkes, J. J. Hartvigsen, M. Shahnam, Int. J. Hydrogen Energy **2007**, *32*, 440 –450.

[4] F. S. Baumann, J. Fleig, G. Cristiani, B. Stuhlhofer, H.-U. Habermeier, J. Maier, J. Electrochem. Soc. **2007**, *154*, B931 –B941.

[5] S. B. Adler, Chem. Rev. **2004**, *104*, 4791 – 4844.

[6] S. B. Adler, J. A. Lane, B. C. H. Steele, J. Electrochem. Soc. **1996**, *143*, 3554 – 3564.

[7] J. Fleig, J. Power Sources **2002**, *105*, 228 – 238.

[8] W. C. Chueh, Y. Hao, W. Jung, S. M. Haile, Nat. Mater. **2012**, *11*, 155 – 161.

[9] A. Bieberle, L. P. Meier, L. J. Gauckler, J. Electrochem. Soc. **2001**, *148*, A646 – A656.

[10] J. Mizusaki, H. Tagawa, T. Saito, K. Kamitani, T. Yamamura, K. Hirano, S. Ehara, T. Takagi, T. Hikita, M. Ippommatsu, S. Nakagawa, K. Hashimoto, J. Electrochem. Soc. **1994**, *141*, 2129 – 2134.

[11] D. E. Starr, Z. Liu, M. Hvecker, A. Knop-Gericke, H. Bluhm, Chem. Soc. Rev. **2013**, *42*, 5833 – 5857.

[12] E. J. Crumlin, H. Bluhm, Z. Liu, J. Electron Sprectrosc. **2013**, *190* PartA, 84 – 92.

[13] C. Rameshan, et al., Angew. Chem. Int. Ed. **2010**, *49*, 3224 – 3227; Angew. Chem. **2010**, *122*, 3292 – 3296.

[14] E. J. Crumlin, E. Mutoro, W. T. Hong, M. D. Biegalski, H. M. Christen, Z. Liu, H. Bluhm, Y. Shao-Horn, J. Phys. Chem. C **2013**, *117*, 16087 – 16094.

[15] E. Mutoro, E. J. Crumlin, H. Pçpke, B. Luerssen, M. Amati, M. K. Abyaneh, M. D. Biegalski, H. M. Christen, L. Gregoratti, J. Janek, Y. Shao-Horn, J. Phys. Chem. Lett. **2012**, *3*, 40 – 44.

[16] A.-K. Huber, M. Falk, M. Rohnke, B. Luerssen, L. Gregoratti, M. Amati, J. Janek, Phys. Chem. Chem. Phys. **2012**, *14*, 751 – 758.

[17] S. C. DeCaluwe, M. E. Grass, C. Zhang, F. E. Gabaly, H. Bluhm, Z. Liu, G. S. Jackson, A. H. McDaniel, K. F. McCarty, R. L. Farrow,M. A. Linne, Z. Hussain, B.W. Eichhorn, J. Phys. Chem. C **2010**, *114*, 19853 – 19861.

[18] C. Zhang, et al., J. Am. Chem. Soc. **2013**, *135*, 11572 – 11579.

[19] W. C. Chueh, A. H. McDaniel, M. E. Grass, Y. Hao, N. Jabeen, Z. Liu, S. M. Haile, K. F. McCarty, H. Bluhm, F. El Gabaly, Chem. Mater. **2012**, *24*, 1876 – 1882.

[20] M. V. Patrakeev, J. A. Bahteeva, E. B. Mitberg, I. A. Leonidov, V. L. Kozhevnikov, K. R. Poeppelmeier, J. Solid State Chem. **2003**, *172*, 219 – 231.

[21] M. Kuhn, S. Hashimoto, K. Sato, K. Yashiro, J. Mizusaki, Solid State Ionics **2011**, *195*, 7 – 15.

[22] A. Nenning, A. K. Opitz, T. Huber, J. Fleig, Phys. Chem. Chem. Phys. **2014**, *16*, 22321 – 22336.

[23] Innovative Station for In Situ Spectroscopy, https://www.helmholtz-berlin.de/pubbin/igama_output?modus=einzel&sprache=en&gid=1607&typoid=50740, accessed **2014**, Sept. 24th.

[24] J. M. Haag, S. A. Barnett, J. W. Richardson, K. R. Poeppelmeier, Chem. Mater. **2010**, *22*, 3283 – 3289.

[25] D. Neagu, G. Tsekouras, D. N. Miller, H. Ménard, J. T. S. Irvine, Nat. Chem. **2013**, *5*, 916 – 923.

[26] M. B. Katz, G.W. Graham, Y. Duan, H. Liu, C. Adamo, D. G. Schlom, X. Pan, J. Am. Chem. Soc. **2011**, *133*, 18090 – 18093.

[27] F. Tonus, M. Bahout, P. D. Battle, T. Hansen, P. F. Henry, T. Roisnel, J. Mater. Chem. **2010**, *20*, 4103 – 4115.

[28] M. Sase, K. Yashiro, K. Sato, J. Mizusaki, T. Kawada, N. Sakai, K. Yamaji, T. Horita, H. Yokokawa, Solid State Ionics **2008**, *178*, 1843 – 1852.

[29] Y. Chen, Z. Cai, Y. Kuru, W. Ma, H. L. Tuller, B. Yildiz, Adv. Energy Mater. **2013**, *3*, 1221 – 1229.

Weiterführende Information:

1. Experimentelles

1.1 Probenpräparation

Die LSF-Arbeitselektroden mit darunterliegenden Platin-Stromabnehmern wurden wie folgt hergestellt: 5 nm Ti (BAL-TEC, Deutschland) und 100 nm Pt (99,95% rein, ÖGUSSA, Österreich) wurden durch Sputtern auf Yttrium-stabilisiertem Zirkoniumdioxid (YSZ) abgeschieden (YSZ-Einkristalle: (100)-orientiert, 9,5 mol% Y_2O_3; Anbieter: CrysTec, Deutschland) und anschließend durch Fotolithographie und Argon-Ionenstrahlätzen mikrostrukturiert. Mittels gepulster Laser Deposition (PLD) mit einem 248 nm Kr-F-Excimerlaser (Compex Pro 201F, Coherent Lambda, Deutschland) wurden auf die aufgebrachten Stromsammler LSF Dünnschichten abgeschieden; Laserenergie und Repetitionsrate waren jeweils 400 mJ und 5 Hz. Der Sauerstoffhintergrunddruck in der PLD-Kammer war 4×10^{-2} mbar, die Substrattemperatur war 650 °C (mittels Pyrometer gemessen, Heitronics, Deutschland), die Beschichtungszeit von 30 min führte zu einer Filmdicke von etwa 300 nm. Die resultierende aktive Fläche der LSF Dünnfilmelektroden war 0,111 cm^2. Das LSF Target wurde aus $La_{0.6}Sr_{0.4}FeO_{3-\delta}$ Pulver (Sigma-Aldrich) durch isostatisches Pressen und Sintern bei 1200 °C hergestellt. Eine Skizze und eine mikroskopische Aufnahme einer LSF Arbeitselektrode mit darunterliegenden Stromsammlern sind im Hauptartikel in Abb. 1a und b dargestellt.

Die porösen Gegenelektroden wurden vor der Präparation der Arbeitselektrode hergestellt. Dies erfolgte durch Aufbringen von LSF Paste ($La_{0.6}Sr_{0.4}FeO_{3-\delta}$ Pulver, Ethylcellulose und α-Terpineol; alle Sigma-Aldrich) und anschließend durch Aufbringen von Pt-Paste (Gwent Electronics, UK), gefolgt von Trocknen und Tempern für 5 Stunden bei 850 °C. Es soll hier betont werden, dass der gemessene Polarisationswiderstand der Gegenelektrode bei 620 °C ca. 8 Ω beträgt, was deutlich kleiner ist als der Widerstand der Dünnfilm-Arbeitselektrode (siehe unten). Somit kann eine Spannungsabfall an der Gegenelektrode vernachlässigt werden (zur Erklärung: die poröse Elektrode agiert gleichzeitig als Gegenelektrode und als reversible Referenzelektrode). Die Polarisation der Arbeitselektrode kann daher aus Zweipunkt-messungen durch Abziehen des ohmschen Spannungsabfalls im YSZ-Elektrolyten (und den kontaktierenden Drähten) von der angelegten Spannung bestimmt werden; Einzelheiten zu der Berechnung der Überspannung sind in Abschnitt 2.1 erläutert.

1.2 Umgebungsdrucknahe XPS-Experimente und gleichzeitige Impedanz-spektroskopische Messungen unter elektrochemischer Polarisation

Die Experimente wurden am ISISS Strahlrohr des HZB/BESSY II Synchrotron in Berlin[1] an der neuen „near-ambient pressure high energy XPS“ (NAP-HE-XPS) Endstation durchgeführt. Dieses Setup ermöglicht XPS und XAS-Messungen bei hohen Drücken (bis 20 mbar, typischerweise 1 mbar) im Spektralbereich von weicher bis harter Röntgenstrahlung. Die Hauptbestandteile sind eine Hochdruckzelle mit einem angebauten differentiell gepumpten Halbkugelanalysator (Phoibos 150 Plus, SPECS) mit einem 2D-delay line Detektor. Eine allgemeine sowie eine detailliertere Beschreibung des NAP-HE-XPS Setups ist in der Literatur zu finden[2-4].

Der schematische Aufbau einer Probe, die für die in-situ XPS Experimente unter elektrochemischer Polarisation und die gleichzeitig durchgeführten Impedanz Messungen verwendet wurde, ist in Abb. 1c des Haupttextes dargestellt. Die LSF Arbeitselektrode wurde mit dem Pluspol des Impedanz-Analysators/Potentiostaten verbunden, die poröse Gegenelektrode mit dem geerdeten Kontakt. Die Proben wurden durch Bestrahlung des Pt-Rückblechs mit einem Infrarotlaser geheizt. Die Temperatur wurde sowohl durch Messen der MIEC Oberflächentemperatur mittels Pyrometer (IMPAC IGAR12-LO) bestimmt, sowie aus den elektrochemischen Impedanzmessungen über die Leitfähigkeit des YSZ Elektrolyten[5,6]. Beides ergab Temperaturen von 620 ± 15 °C. Elektrochemische Impedanzmessungen mit und ohne Gleichspannungsanteil wurden mit einem Alpha-A Hochleistungs-Frequenzanalysator, ausgestattet mit einer POT/GAL 30V 2A-Schnittstelle (beide: Novocontrol, Deutschland), durchgeführt. Die Impedanzspektroskopie wurde in einem Frequenzbereich von 10 mHz bis 1 MHz durchgeführt und der Effektivwert der Wechselspannung wurde dabei auf 5 mV beschränkt um eine Peakverbreiterung der XPS-Signale zu vermeiden.

Für die elektrochemische Polarisation wurden Gleichspannungen zwischen +700 mV und -500 mV an die Arbeitselektrode angelegt. Das Anlegen der einzelnen Spannungswerte erfolgte dabei nach dem Zufallsprinzip (einige der eingestellten Spannungen wurden außerdem wiederholt) um die Reversibilität und Reproduzierbarkeit der Strom-Spannungs-Kennlinien und der XPS Ergebnisse zu überprüfen. Bei jeder eingestellten Gleichspannung wurde die Aufzeichnung von XPS-Spektren erst gestartet, nachdem ein stabiler Gleichstromfluss erreicht war. Die oberflächensensitiven Fe2p Spektren wurden mit einer primären Photonenenergie von 845 eV gemessen. Während den simultan durchgeführten elektrochemischen- bzw. XPS-Experimenten wurde der Partialdruck von H_2 und H_2O mit jeweils 0,25 mbar konstant gehalten, womit ein Gesamtdruck von 0,5 mbar in der Kammer

vorlag. Zu beachten ist, dass bei dem gegebenen Setup (beide Elektroden befinden sich in der gleichen Atmosphäre) eine Quantifizierung der erzeugten Wasserstoffmenge nicht möglich ist, da Wasserstoff an einer Elektrode erzeugt wird und gleichzeitig an der anderen wieder verbraucht wird. Daher verändert sich die Gesamtzusammensetzung der Atmosphäre während der elektrochemischen Polarisation der Probe in der NAP-XPS Kammer nicht.

2 Ergebnisse der elektrochemischen Messungen

2.1 Gleichstrommessungen

In Abb. 2 des Haupttextes ist die Strom-Spannungs-Kennlinie einer LSF-Arbeitselektrode gezeigt (Auftragung des gemessenen Gleichstroms gegen die angelegte Überspannung). Die Überspannung η wurde aus der eingestellten Gleichspannung (U_{dc}) durch Subtraktion des ohmschen Abfalls im Elektrolyt und den Anschlussdrähten mittels

$$\eta = U_{dc} - R_{HF} \cdot I_{dc} \qquad (1)$$

berechnet. Dabei bezeichnen R_{HF} den (ohmschen) Hochfrequenz-Achsenabschnitt – gemessen mittels Impedanzspektroskopie (siehe unten) – und I_{dc} den Gleichstrom.

Die Strom-Überspannungs-Kurve in Abb. 2 ist stark asymmetrisch mit einer starken Nichtlinearität bei kathodischer Polarisation und einer viel schwächeren Nichtlinearität im anodischen Bereich. Wegen dieser Asymmetrie war es nicht möglich, für den gesamten Spannungsbereich eine der gängigen Fitfunktionen für Elektroden-Reaktionen (z.B. auf Exponentialfunktionen basierend) mit elektrochemisch sinnvollen Parametern anzuwenden. Um die ungewöhnliche Form der gemessenen Kurve zu veranschaulichen, nahmen wir ladungstransferlimitierte Elektrodenkinetik an, wobei die Strom-Überspannung Kurve mit der Butler-Volmer (B-V) Gleichung beschrieben werden kann.

$$I_{dc} = I_0 \cdot \left(e^{\frac{\alpha z e_0}{k_B T}\eta} - e^{\frac{(\alpha-1) z e_0}{k_B T}\eta} \right) \qquad (2)$$

Darin ist I_0 der Austauschstrom, α der Symmetriefaktor (wobei α = 0.5 eine symmetrische Aktivierungsbarriere des geschwindigkeitsbestimmenden Ladungstransferschritts bedeutet),

und z die Anzahl der übertragenen Ladungen; e_0, k_B, und T stehen jeweils für Elementarladung, Boltzmann-Konstante und die absolute Temperatur. Einen Vergleich der Messdaten und Simulationsergebnisse zeigt Abb. s1. Der Strom bei stark anodischer und stark kathodischer Polarisation kann nur mit erheblich unterschiedlichen i_0 und z Werten in der B-V-Simulation beschrieben werden. Der Teil der Kurve zwischen 0 und -100 mV kann jedoch mit diesen Funktionen überhaupt nicht erklärt werden. Nur unter der Annahme von eher unrealistischen Parametern ($1-\alpha = 0.85$, $z = 2$) kann dieser Teil der I-η Beziehung simuliert werden (aber die gemessenen Ströme bei hohen Polarisationen können damit nicht erklärt werden). Daraus kann gefolgert werden, dass der steile Anstieg des Stroms bei kathodischer Polarisation über -20 mV ein starkes Indiz für eine grundlegende Änderung des Reaktionsmechanismus an der Elektrodenoberfläche ist.

Außerdem sei hervorgehoben, dass die Strom-Spannungs-Kurve in Abb. 2 – abgesehen von einer kleinen Veränderung unmittelbar nach dem ersten Heizen der Probe – reproduzierbar gemessen werden konnte. (Dies gilt auch für den Oberflächenwiderstand der Elektrode welcher durch Impedanzspektroskopie gemessen wurde. Auch für diesen konnte während der ersten Messungen nur eine geringfügige Verschlechterung des Oberflächenwiderstands beobachtet werden.) Irreversible Veränderungen der Elektrode während der elektrochemischen Experimente können so als Ursprung der asymmetrisch geformten Kurven ausgeschlossen werden.

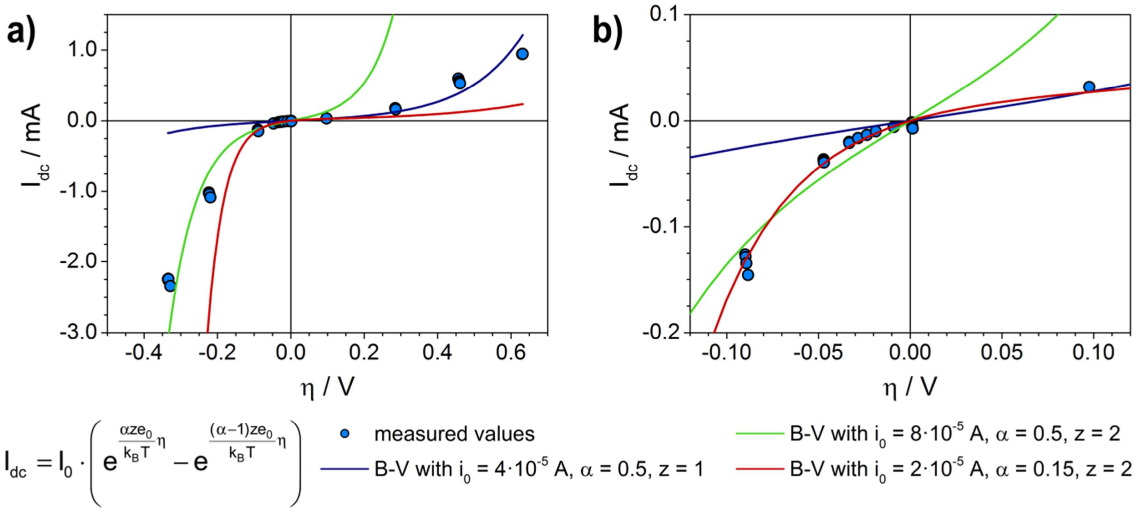

$$I_{dc} = I_0 \cdot \left(e^{\frac{\alpha z e_0}{k_B T}\eta} - e^{\frac{(\alpha-1) z e_0}{k_B T}\eta} \right)$$

Abbildung s1: *Vergleich einer gemessenen Strom-Überspannungs Kurve mit Simulationen auf Basis der Butler-Volmer-Gleichung. a) Gesamter Spannungsbereich. b) Vergrößerung des Bereichs moderater Polarisation von (a). Es wird deutlich, dass die gesamte Kurve mittels klassischer Butler-Volmer-Kinetik nicht erklärt werden kann.*

2.2 Impedanz Spektroskopie

Typische Impedanzspektren im elektrochemischen Gleichgewicht sowie unter elektrochemischer Polarisation sind in Abb. s2 gezeigt. Qualitativ zeigt jedes der Spektren gleiche Merkmale: (i) einen hochfrequenten Z_{re}-Achsenabschnitt, (ii) eine relativ gut abgetrennte (halbkreisförmige) Schulter im Hochfrequenzbereich, (iii) ein zusätzliche Schulter im mittleren Frequenzbereich, und (iv) einen geringfügig abgeflachten Niederfrequenz Halbkreis. Der Hochfrequenz-Achsenabschnitt (R_{HF}) kann vor allem dem Widerstand der Ionenleitung in YSZ [7] und einem ca. 5 Ω Widerstand der Messleitungen und Kontakte zugeordnet werden. Die Elektrodenpolarisation (Gleichstrom-Messungen) wurde um die ohmschen Verluste korrigiert, welche durch diesen Hochfrequenz-Offset-Widerstand (R_{HF}) verursacht werden – siehe oben. Die sehr kleine halbkreisförmige Hochfrequenz-Schulter wird sehr wahrscheinlich durch einen Ionenübergangswiderstand an der Elektroden|Elektrolyt Grenzfläche und eine dementsprechende Grenzflächenkapazität[7] verursacht; teilweise könnte auch die Gegenelektrode zu diesem Impedanz-Merkmal beitragen. In Übereinstimmung mit einer ähnlichen Studie auf $SrTi_{0.7}Fe_{0.3}O_{3-\delta}$ Elektroden[8] können die Schulter bei mittleren Frequenzen und der Niederfrequenz Bogen, jeweils dem Elektronentransport innerhalb des MIEC Dünnfilms und der elektrochemischen Reaktion an der MIEC Oberfläche, zugeordnet werden. Die involvierte Kapazität ist die chemische Kapazität des gemischt leitenden Elektrodenmaterials LSF.[9] In all den hier betrachteten Fällen dominiert der Niederfrequenzhalbkreis, und damit der Widerstand der Oberflächenreaktion, den Polarisationswiderstand der LSF Elektrode. Daraus können wir schließen, dass für die in dieser Studie verwendete Geometrie, die Polarisation der Arbeitselektrode praktisch homogen ist. Im Falle einer homogen polarisierten gemischtleitenden Elektrode mit oberflächenlimitierter Kinetik wird die Überspannung η direkt in eine Änderung des chemischen Potentials von Sauerstoff in der (Festkörper-) Elektrode $\mu(O_2)_{MIEC}$ „übersetzt" und es gilt daher $\Delta\mu(O_2)_{Oberfläche} = \mu(O_2)_{MIEC} - \mu(O_2)_{Gas} = 4e_0\eta$. Somit kann die thermodynamische Triebkraft für die Oberflächenreaktion $\Delta\mu(O_2)_{Oberfläche}$ durch angelegen einer entsprechenden Überspannung definiert eingestellt werden.

Wie in Abb. s2 gezeigt wird der Durchmesser der Niederfrequenzhalbkreis durch sowohl anodische als auch kathodische Gleichspannung verringert. Diese Spannungsabhängigkeit der differentiellen Impedanz passt sehr gut zur nichtlinearen Strom-Spannungs-Kennlinie und ist ein übliches Verhalten von Elektrodenreaktionen. Ungewöhnlich ist jedoch die sehr starke Spannungsabhängigkeit die schon bei sehr kleinen kathodischen Polarisationen gefunden

wurde, siehe -47 mV in Abb. s2. Für die Auswertung des Widerstands der elektrochemischen Reaktion an der Elektrodenoberfläche wurde nur der niedrige Frequenzteil des Spektrums (d.h. nur der Niederfrequenzbogen) mithilfe der im Inset von Abb. s2a gezeigten Schaltung ausgewertet. In diesem Ersatzschaltbild werden jeweils die Sauerstoffaustauschreaktion auf der LSF Oberfläche und die chemische Kapazität des LSF durch den Widerstand $R_{Oberfläche}$ und das Element konstanter Phase[10] CPE repräsentiert. Die Serienwiderstand R_{offset} steht für alle resistiven Beiträge mit höheren Relaxationsfrequenzen und ist damit größer als R_{HF}. Obwohl dieses Ersatzschaltbild die Impedanz der untersuchten Elektroden stark vereinfacht, ist im Falle einer homogenen elektrochemischen Polarisation eine vernünftige Abschätzung des dominierenden Oberflächenwiderstands $R_{Oberfläche}$ möglich.[8]

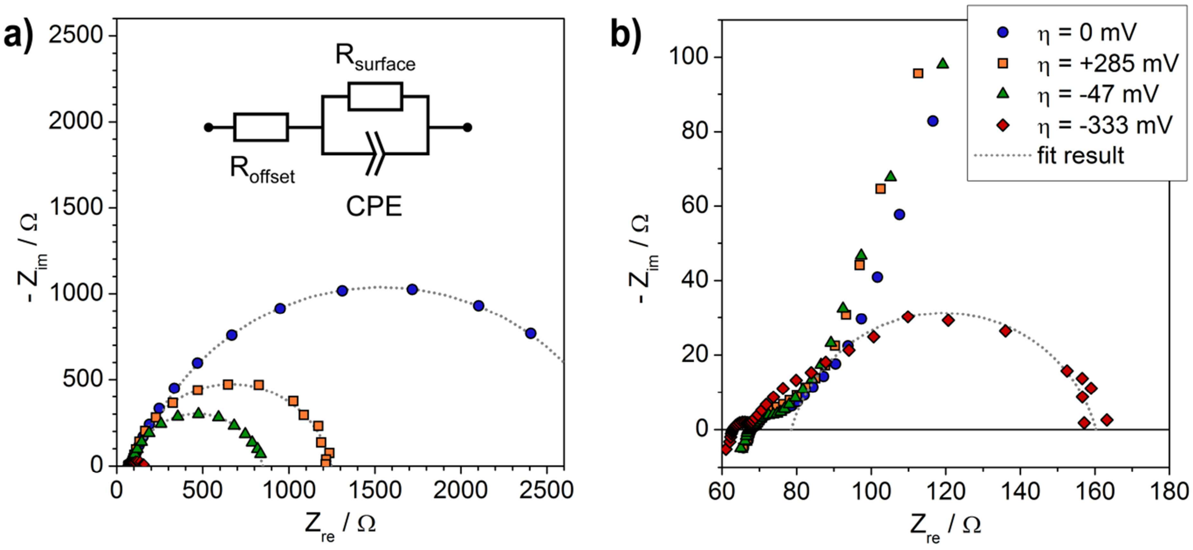

Abbildung s2: *a) Impedanzspektren (Nyquist Plot) gemessen bei 620 ± 15 °C in 0,25 mbar* H_2 *+ 0,25 mbar* H_2O *Atmosphäre unter Gleichgewichtsbedingungen (Kreise), als auch bei anodischer (Quadrate) und kathodischer (Dreiecke und Rauten) Polarisation. Die gepunkteten Kurven zeigen die Ergebnisse, welche durch Anpassung an das im Inset dargestellte Ersatzschaltbild erhalten wurden. b) Vergrößerung des Spektrums, gemessen bei einer kathodischen Überspannung von -333 mV von (a).*

3 XPS Ergebnisse

3.1 Fe2p und Valenzbandspektren

Fe2p Fotoelektronenspektren, gemessen mit 845 eV Photonenenergie und bei verschiedener elektrochemischer Polarisation, sind als Insets in Abb. 2 des Haupttext dargestellt und Details dazu werden in Abb. S3a gezeigt. Die Informationstiefe dieser NAP-XPS Experimente ist 0.5 ± 0.1 nm (dieser Wert wird durch Berechnung der inelastischen freien Weglänge der Photoelektronen in LSF mithilfe der NIST SR 71 Datenbank und der TPP-2M-Gleichung erhalten). Abhängig von der angelegten elektrochemischen Überspannung können in Abb. s3a zwei grundsätzlich verschiedene Arten von Fe2p Spektren gefunden werden:

(i) zwischen etwa -20 mV und der höchsten anodischen Überspannung können zwei ausgeprägte Peaks bei ca. 710 eV und ca. 724 eV Bindungsenergie (BE) beobachtet werden, welche jeweils $Fe2p_{3/2}$ und $Fe2p_{1/2}$ zugeordnet werden können. Beide Bindungsenergien sind in guter Übereinstimmung mit Literaturwerten für Fe^{+II} und/oder Fe^{+III}.[11] Ein $Fe2p_{3/2}$-Satellit bei ca. 716 eV ist im Spektrum, welches ohne elektrochemische Polarisation gemessen wurde, schwach sichtbar und entspricht einer Fe^{+II} Spezies. Dies lässt auf eine beträchtliche Menge an Fe^{+II} an der Oberfläche schließen, während volumensensitive Studien auf $La_{0.6}Sr_{0.4}FeO_{3-\delta}$[12] meist die Präsenz von Fe^{+III} zeigen. Eine hohe Reduzierbarkeit von Oxidoberflächen wurde bereits für ceroxidbasierte Anoden[13] festgestellt, und unterstreicht die Bedeutung von in-situ Untersuchungen.[14] (Die Bindungsenergie eines zusätzlichen Signals bei ca. 730 eV gehört prinzipiell zum Satellit von $Fe2p_{1/2}$. Aufgrund der hohen Intensität und relativ scharfen Peakform können hier zusätzliche Beiträge – z.B. von Auger-Linien – allerdings nicht ausgeschlossen werden.)

(ii) Zusätzlich zu den oben beschriebenen Signalen, werden bei kathodischen Überspannungen negativer als -20 mV zwei weitere Peaks bei etwa 707 eV und 720 eV beobachtet. Diese Peaks können jeweils $Fe2p_{3/2}$ und $Fe2p_{1/2}$ von metallischem Eisen zugeordnet werden.[11] Es wird darauf hingewiesen, dass durch Veränderung der Messposition Strahlschädigungen des LSF ausgeschlossen wurden (und dass damit ausgeschlossen werden kann, dass die gezeigten chemischen Veränderungen während der XPS Experimente von Strahlenschäden stammen).

XPS Valenzbandspektren von LSF wurden mit einer Photonenenergie von 140 eV aufgenommen (die Photonenenergie wurde so gewählt, dass die kinetische Energie der emittierten Photoelektronen die gleiche ist wie für die Fe2p Spektren; damit ist die Informationstiefe der Messungen identisch); dies erfolgte bei gleichen elektrochemischen

Polarisationen wie die Fe2p Spektren – siehe Abb. s3b. Interessanterweise zeigen jene Messungen unter kathodischer Polarisation, bei denen die Fe^{0} Peaks in den Fe2p Spektren auftauchen, auch die Ausbildung einer Fermikante im Bereich sehr geringer Bindungsenergie (siehe Pfeile in Abb. s3b). Die Korrelation dieser beiden Änderungen in den XPS-Spektren kann als starker Beweis für die oberflächennahe Bildung einer metallischen Fe-Phase interpretiert werden. Es soll betont werden, dass die Entwicklung einer metallischen Fe Spezies auf der Elektrodenfläche nicht zu einer irreversiblen Zersetzung der LSF Elektrode geführt hat. Vielmehr wurde ein (überraschend) reversibles Verhalten in den elektrochemischen- und XPS-Versuchen gefunden (siehe Fig. 2 und 3a des Haupttexts).

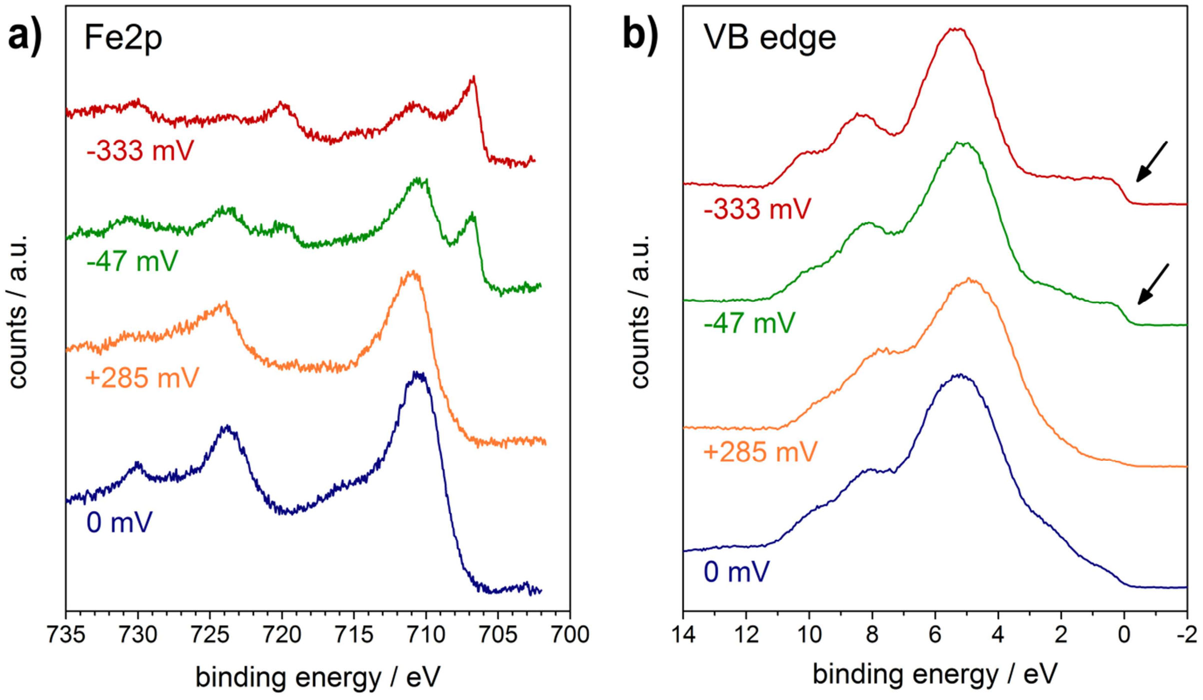

Abbildung s3: *a) Fe2p XPS-Spektren gemessen mit 845 eV Photonenenergie; alle Spektren sind gleich skaliert und nur versetzt zueinander aufgetragen. Die Bildung von metallischem Fe und eine starke Abnahme des Gesamt Fe-Signals sind unter kathodischer Polarisation deutlich sichtbar. Die angelegten Überspannungen sind für jede Kurve angegeben. b) Valenzbandenspektren gemessen mit 140 eV Photonenenergie; alle Spektren sind gleich skaliert und nur versetzt zueinander aufgetragen. Die angelegte Überspannung ist bei jeder Kurve angegeben. Die Fermi Kanten, die bei kathodischer Polarisation auftauchen, sind durch Pfeile angezeigt.*

3.2 Sr3d, La3d und O1s Spektren

Neben Fe2p Spektren wurden bei jeder elektrochemischen Polarisation auch La3d, Sr3d und O1s Spektren aufgezeichnet – sie werden in Abb. s4 gezeigt. Es sind jedoch nur geringfügige Änderungen in diesen XPS-Spektren zu sehen, die bei weitem nicht so ausgeprägt sind, wie im Falle der Fe2p und Valenzbandspektren. Abgesehen von einigen geringfügigen Verschiebungen der Bindungsenergie (welche möglicherweise ihren Ursprung in einer unzureichenden Bindungsenergiekorrektur – wegen der fehlenden Fermikante – haben) wurden keine signifikanten Veränderungen in den La3d, Sr3d und O1s Spektren gefunden. Diese Beobachtungen stützten die Annahme, dass das metallische Eisen, welches unter kathodischer Polarisation gebildet wird, nicht Bestandteil der Perowskitphase ist. Ansonsten wäre eine viel deutlichere Änderung der La3d, Sr3d und O1s Spektren zu erwarten.

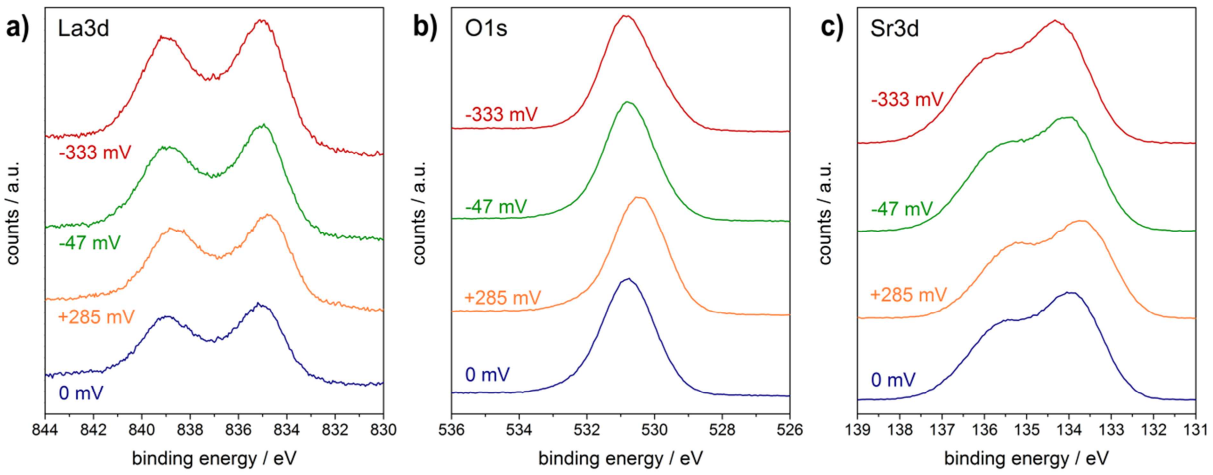

Abbildung s4: *a) La3d XPS-Spektren gemessen mit 975 eV Photonenenergie; alle Spektren sind gleich skaliert und nur versetzt zueinander aufgetragen. Die angelegte Überspannung ist bei der jeweiligen Kurve angegeben. b) O1s XPS-Spektren gemessen mit 650 eV Photonenenergie; alle Spektren sind gleich skaliert und nur versetzt zueinander aufgetragen und die jeweiligen Polarisationen sind bei den Kurven angegeben. c) Sr3d XPS Spektren, gemessen mit 252 eV Photonenenergie; alle Spektren sind gleich skaliert und nur versetzt zueinander aufgetragen und die jeweiligen Polarisationen sind bei den Kurven angegeben.*

Oberflächennahe Sr3d Spektren, welche auf (La,Sr)$CoO_{3-\delta}$ (LSC) Elektroden in Sauerstoff Atmosphäre unter elektrochemischer Polarisation gemessen wurden, sind in Referenz [15] beschrieben. In der zitierten Studie wurden zwei Sr Spezies – eine bei niedrigerer und eine bei

höherer BE – beobachtet und jeweils als Gitter- bzw. Oberflächenspezies interpretiert. In ähnlicher Weise kann die Form unserer Spektren in reduzierender Atmosphäre durch den Beitrag von zwei Sr-Spezies (vgl. Abb. s4c) erklärt werden. Jedoch geht eine quantitative Analyse der Spektren (außer für Fe) über den Rahmen der vorliegenden Arbeit hinaus.

3.3 Auswertung der Fe2p XPS Daten

Fe2p Spektren wurden mit Hilfe eines vereinfachten Peakmodells gefittet (Software: CasaXPS), um die relativen Anteile von metallischem und oxidischem Eisen zu quantifizieren. Daher wurde nur das $Fe2p_{3/2}$ Signal für die Auswertung herangezogen. Für die metallischen und oxidischen Anteile wurde eine asymmetrische Lorenz-Linienform verwendet – allgemein als LF-Linienform bezeichnet.[16] Diese Linienform hat einen exponentiellen Abfall auf der Seite der hohen Bindungsenergien um der Asymmetrie Rechnung zu tragen. Die Peak-Parameter LF(1.3,2,10,0) und LF(0.8,2,15,0) wurden jeweils für die $Fe^{+II/III}$ und die Fe^{0} Peaks verwendet. Die Autoren sind sich bewusst, dass diese Methode der Peakanalyse eine starke Vereinfachung der tatsächlich sehr komplexen Fe-Peakstruktur ist.[17, 18] Jedoch ist dieses Modell aufgrund der geringen Anzahl an Fitparametern und Beschränkungen sehr gut geeignet, um Peakbeiträge von metallischem und oxidischem Eisen zu trennen.

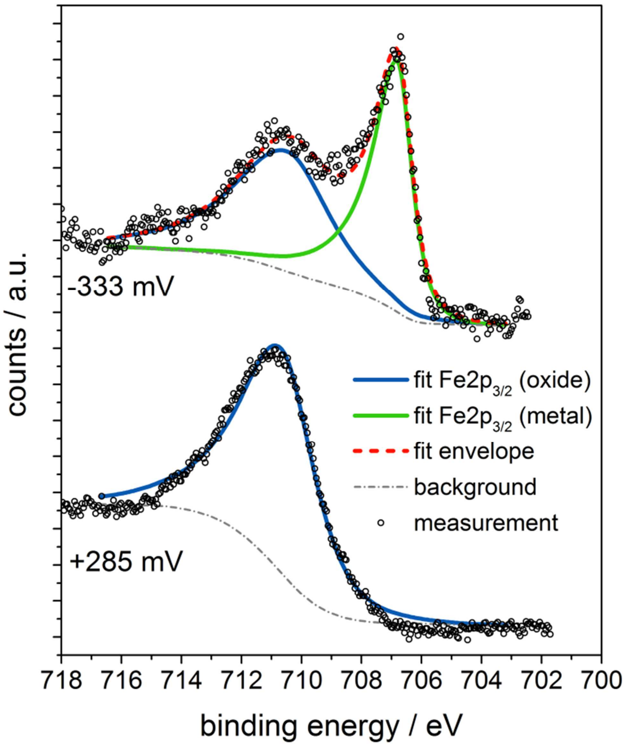

Abbildung s5: *Fit der $Fe2p_{3/2}$-Spektren bei Überspannungen von -333 mV und +285 mV; die Intensitätsskala der beiden Spektren wurde zu besseren Visualisierung angepasst.*

Ein Peakfit der $Fe2p_{3/2}$-Spektren, gemessen bei -333 mV und +285 mV, ist in Abb. s5 dargestellt. Er zeigt die ausgeprägten Unterschiede zwischen den Oberflächen von anodisch und kathodisch polarisiertem LSF. Für diese Peakfits wurden eine metallische (Fe^0) und eine oxidische Eisenspezies (Fe^{+II} und/oder Fe^{+III}) berücksichtigt. Die resultierende Bindungsenergie des $Fe2p_{3/2}$ Peaks für die oxidische Fe Spezies ist 710,3 ± 0,2 eV und jene für die metallische Fe Spezies ist 706,8 ± 0,1 eV. Diese Ergebnisse stehen in guter Übereinstimmung mit Literaturdaten.[11] Die Quantifizierung der relativen Menge von Fe^{+II} bzw. Fe^{+III} ist jedoch nicht eindeutig möglich. Dies ist zum Teil auf die Energiekalibrierung der XPS Spektren zurückzuführen, die nur mithilfe der Valenzbandkante als Bezugspunkt möglich war (bitte beachten: der Fehler, der durch die Bewegung des Röntgen Monochromators verursacht wird, ist ca. ± 0,15 eV).

Referenzen:

[1] Innovative Station for In Situ Spectroscopy, https://www.helmholtz-berlin.de/pubbin/igama_output?modus=einzel&sprache=en&gid=1607&typoid=50740 accessed 2014, Sept. 24th.

[2] H. Bluhm, M. Hävecker, A. Knop-Gericke, E. Kleimenov, R. Schlögl, D. Teschner, V. I. Bukhtiyarov, D. F. Ogletree, M. Salmeron, *The Journal of Physical Chemistry B* **2004**, *108*, 14340-14347.

[3] A. Knop-Gericke, E. Kleimenov, M. Hävecker, R. Blume, D. Teschner, S. Zafeiratos, R. Schlögl, V. I. Bukhtiyarov, V. V. Kaichev, I. P. Prosvirin, A. I. Nizovskii, H. Bluhm, A. Barinov, P. Dudin, M. Kiskinova, in *Advances in Catalysis, Vol. Volume 52* (Eds.: C. G. Bruce, K. Helmut), Academic Press, **2009**, pp. 213-272.

[4] ISISS Station, https://www.helmholtz-berlin.de/pubbin/igama_output?modus=einzel&sprache=en&gid=1671, accessed 2014 Sept. 24th.

[5] A. K. Opitz, J. Fleig, *Solid State Ionics* **2010**, *181*, 684–693.

[6] A. K. Opitz, A. Lutz, M. Kubicek, F. Kubel, H. Hutter, J. Fleig, *Electrochimica Acta* **2011**, *56*, 9727– 9740.

[7] F. S. Baumann, J. Fleig, H. U. Habermeier, J. Maier, *Solid State Ionics* **2006**, *177*, 1071-1081.

[8] A. Nenning, A. K. Opitz, T. Huber, J. Fleig, *Phys. Chem. Chem. Phys.* **in press**.

[9] J. Jamnik, J. Maier, *Physical Chemistry Chemical Physics* **2001**, *3*, 1668-1678.

[10] J. Fleig, *Solid State Ionics* **2002**, *150*, 181-193.

[11] M. Descostes, F. Mercier, N. Thromat, C. Beaucaire, M. Gautier-Soyer, *Applied Surface Science* **2000**, *165*, 288-302.

[12] M. Kuhn, S. Hashimoto, K. Sato, K. Yashiro, J. Mizusaki, *Solid State Ionics* **2011**, *195*, 7-15.

[13] W. C. Chueh, A. H. McDaniel, M. E. Grass, Y. Hao, N. Jabeen, Z. Liu, S. M. Haile, K. F. McCarty, H. Bluhm, F. El Gabaly, *Chemistry of Materials* **2012**, *24*, 1876-1882.

[14] C. Rameshan, W. Stadlmayr, C. Weilach, S. Penner, H. Lorenz, M. Hävecker, R. Blume, T. Rocha, D. Teschner, A. Knop-Gericke, R. Schlögl, N. Memmel, D. Zemlyanov, G. Rupprechter, B. Klötzer, *Angewandte Chemie International Edition* **2010**, *49*, 3224-3227.

[15] E. J. Crumlin, E. Mutoro, W. T. Hong, M. D. Biegalski, H. M. Christen, Z. Liu, H. Bluhm, Y. Shao-Horn, *The Journal of Physical Chemistry C* **2013**, *117*, 16087-16094.

[16] Lorentzian Asymmetric Lineshape, http://www.casaxps.com/help_manual/manual_updates/LA_Lineshape.pdf, accessed 2014, July, 2nd.

[17] M. Aronniemi, J. Sainio, J. Lahtinen, *Surf Sci* **2005**, *578*, 108-123.

[18] M. C. Biesinger, B. P. Payne, A. P. Grosvenor, L. W. M. Lau, A. R. Gerson, R. S. C. Smart, *Applied Surface Science* **2011**, *257*, 2717-2730.

Im Haupttext abgekürzte Refernzen:

C. Rameshan, W. Stadlmayr, C. Weilach, S. Penner, H. Lorenz, M. Hävecker, R. Blume, T. Rocha, D. Teschner, A. Knop-Gericke, R. Schlögl, N. Memmel, D. Zemlyanov, G. Rupprechter, B. Klötzer, *Angew. Chem.* **2010**, *122*, 3292-3296; *Angew. Chem. Int. Ed.* **2010**, *49*, 3224-3227.

C. Zhang, Y. Yu, M. E. Grass, C. Dejoie, W. Ding, K. Gaskell, N. Jabeen, Y. P. Hong, A. Shavorskiy, H. Bluhm, W.-X. Li, G. S. Jackson, Z. Hussain, Z. Liu, B. W. Eichhorn, *J. Am. Chem. Soc.* **2013**, *135*, 11572-11579.
